# Transcriptional programs mediating neuronal toxicity and altered glial–neuronal signaling in a *Drosophila* knock-in tauopathy model

**DOI:** 10.1101/gr.278576.123

**Published:** 2024-04

**Authors:** Hassan Bukhari, Vanitha Nithianandam, Rachel A. Battaglia, Anthony Cicalo, Souvarish Sarkar, Aram Comjean, Yanhui Hu, Matthew J. Leventhal, Xianjun Dong, Mel B. Feany

**Affiliations:** 1 Department of Pathology, Brigham and Women's Hospital, Harvard Medical School, Boston, Massachusetts 02115, USA;; 2 Aligning Science Across Parkinson's (ASAP) Collaborative Research Network, Chevy Chase, Maryland 20815, USA;; 3 Genomics and Bioinformatics Hub, Brigham and Women's Hospital, Boston, Massachusetts 02115, USA;; 4 Department of Neurology, Brigham and Women's Hospital, Harvard Medical School, Boston, Massachusetts 02115, USA;; 5 Department of Genetics, Blavatnik Institute, Harvard Medical School, Boston, Massachusetts 02115, USA;; 6 Department of Biological Engineering, Massachusetts Institute of Technology, Cambridge, Massachusetts 02139, USA;; 7 MIT Ph.D. Program in Computational and Systems Biology, Cambridge, Massachusetts 02139, USA

## Abstract

Missense mutations in the gene encoding the microtubule-associated protein TAU (current and approved symbol is MAPT) cause autosomal dominant forms of frontotemporal dementia. Multiple models of frontotemporal dementia based on transgenic expression of human *TAU* in experimental model organisms, including *Drosophila*, have been described. These models replicate key features of the human disease but do not faithfully recreate the genetic context of the human disorder. Here we use CRISPR-Cas-mediated gene editing to model frontotemporal dementia caused by the TAU P301L mutation by creating the orthologous mutation, P251L, in the endogenous *Drosophila tau* gene. Flies heterozygous or homozygous for Tau P251L display age-dependent neurodegeneration, display metabolic defects, and accumulate DNA damage in affected neurons. To understand the molecular events promoting neuronal dysfunction and death in knock-in flies, we performed single-cell RNA sequencing on approximately 130,000 cells from brains of Tau P251L mutant and control flies. We found that expression of disease-associated mutant *tau* altered gene expression cell autonomously in all neuronal cell types identified. Gene expression was also altered in glial cells, suggestive of non-cell-autonomous regulation. Cell signaling pathways, including glial–neuronal signaling, were broadly dysregulated as were brain region and cell type–specific protein interaction networks and gene regulatory programs. In summary, we present here a genetic model of tauopathy that faithfully recapitulates the genetic context and phenotypic features of the human disease, and use the results of comprehensive single-cell sequencing analysis to outline pathways of neurotoxicity and highlight the potential role of non-cell-autonomous changes in glia.

The neuronal microtubule–associated protein TAU (current and approved symbol is MAPT) forms insoluble deposits termed neurofibrillary tangles and neuritic threads in neuronal soma and processes in a diverse group of age-dependent neurodegenerative diseases, including Alzheimer's disease and frontotemporal dementia. These disorders have collectively been termed “tauopathies” (Feany and Dickson 1996; Goedert 2004; Götz et al. 2019). Although wild-type TAU is deposited in Alzheimer's disease and other more common tauopathies, missense mutations in *TAU* occur in rarer familial forms of tauopathy, causing neurodegeneration and insoluble TAU deposition. Autosomal dominant disease-causing mutations occur throughout the TAU protein but are particularly frequent in exon 10, which contains one of four microtubule binding repeats (Ghetti et al. 2015). These repeats mediate microtubule (Lee et al. 1989; Butner and Kirschner 1991) and actin (Cabrales Fontela et al. 2017) binding and are important determinants of TAU aggregation (von Bergen et al. 2000). Experimental models of tauopathy have been created in diverse model organisms, from yeast to non-human primates, by expressing wild-type or frontotemporal dementia–associated mutant forms of human TAU in transgenic animals. Mutant forms of TAU are typically more toxic than wild-type TAU in transgenic model organisms. Work in these models has implicated a number of cellular pathways in mediating TAU neurotoxicity, including mitochondrial dysfunction (Rhein et al. 2009; DuBoff et al. 2012), oxidative stress (Dias-Santagata et al. 2007; Dumont et al. 2011), and aberrant cell-cycle reentry of postmitotic neurons (Andorfer et al. 2005; Khurana et al. 2006).

However, although transgenic models have been useful, they do not faithfully replicate the genetic underpinnings of the authentic human disorders and thus may not allow the identification and study of the full complement of important mediators of tauopathy pathogenesis. We have therefore used CRISPR-Cas9 gene editing to model familial frontotemporal dementia caused by missense mutations in *TAU* more precisely in *Drosophila*. Mutation of proline 301 to leucine in exon 10 is the most common mutation of *TAU* in frontotemporal dementia patients (Poorkaj et al. 2001) and has been frequently modeled in transgenic animals (Goedert and Jakes 2005). The overall structure and expression of TAU are conserved from mammals to *Drosophila* (Heidary and Fortini 2001), with proline 251 being orthologous to human proline 301. We have therefore replaced *Drosophila* Tau proline 251 with leucine (P251L) and phenotypically analyzed the resultant homozygous and heterozygous animals with age. We have additionally performed single-cell sequencing to identify cell populations, networks, and signaling systems altered by mutant *tau* expression.

## Results

### Phenotypic analysis of a *Drosophila* knock-in model of frontotemporal dementia

**Figure 1.**
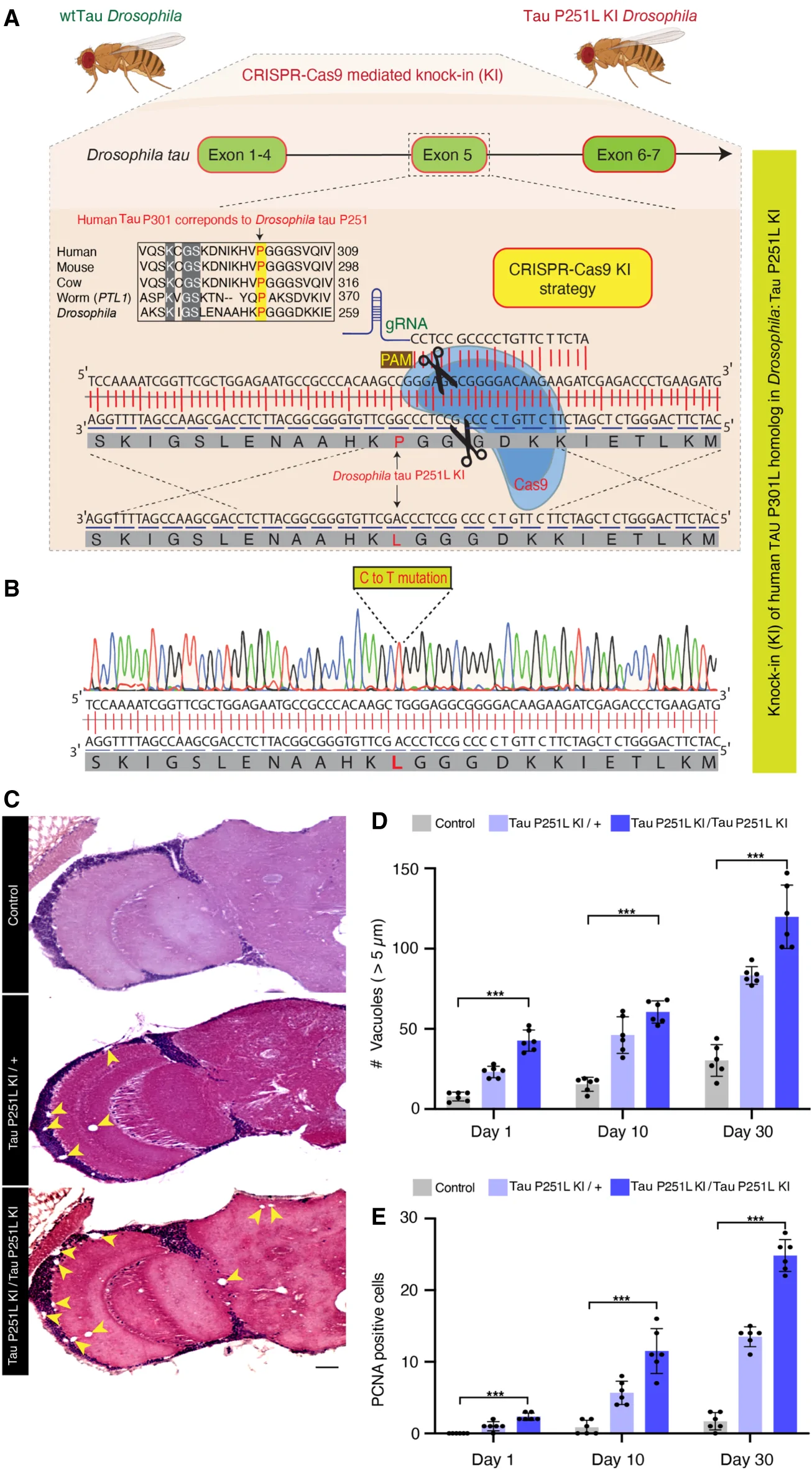
CRISPR-Cas9-mediated knock-in model of frontotemporal dementia in *Drosophila*. (*A*) CRISPR-Cas9 gene editing strategy to knock-in the human TAU P301L homologous mutation in *Drosophila*, Tau P251L, located in exon 5 of *Drosophila tau*. (*B*) Successful mutation in homozygous Tau P251L knock-in flies. (*C*,*D*) Hematoxylin and eosin staining reveals evidence of neurodegeneration as seen by an increased number of brain vacuoles (arrowheads) with age in homozygous and heterozygous knock-in animals. (*C*) Scale bar represents 10 µm. (*E*) Neurodegeneration is accompanied by abnormal cell-cycle reentry as marked by proliferating cell nuclear antigen (PCNA) staining. Flies are 30 d old in *C* and the age indicated in the figure labels in *D*,*E*. (*D*,*E*) n = 6 per genotype and time point. Data are presented as mean ± SD. (***) *P* < 0.001, one-way ANOVA with Tukey post-hoc analysis.

We used CRISPR-Cas9 gene editing to recapitulate the genetic basis of human frontotemporal dementia in the powerful genetic experimental organism *Drosophila* by modeling the disease-causing proline 301 to leucine in fly Tau. Protein sequence alignment shows that the microtubule-binding domains, including human TAU proline 301, are evolutionary conserved from *Drosophila* to humans (Supplemental Fig. S1). The homologous residue of the human TAU proline 301, *Drosophila* Tau proline 251, was mutated to leucine using a highly efficient guide RNA along with single-stranded oligodeoxynucleotides (Fig. 1A,B). Mutant Tau was expressed at equivalent levels to wild-type Tau (Supplemental Fig. S2A).

The expression of frontotemporal dementia-linked forms of mutant TAU, including P301L, leads to age-dependent neuronal loss in patients and in transgenic models (Götz et al. 2001; Lewis et al. 2001; Yoshiyama et al. 2007; Ghetti et al. 2015). We thus examined the histology of brains of heterozygous (P251L/+) and homozygous (P251L) Tau knock-in animals with age. We found increased numbers of cortical and neuropil vacuoles in knock-in animals (Fig. 1C,D). Neurodegeneration in *Drosophila* is frequently accompanied by the formation of brain vacuoles (Heisenberg and Böhl 1979; Buchanan and Benzer 1993; Wittmann et al. 2001; Ordonez et al. 2018). Increasing numbers of vacuoles were observed with advancing age, as well as with two copies of the P251L compared with one copy (Fig. 1C,D). Inappropriate neuronal cycle reentry is a feature of human tauopathy (Husseman et al. 2000) and human *TAU* transgenic animals (Andorfer et al. 2005; Khurana et al. 2006). We stained control and Tau P251L knock-in brains with an antibody directed to proliferating cell nuclear antigen (PCNA) to assess cell-cycle activation (Khurana et al. 2006). We observed increasing cell-cycle reentry with age in Tau P251L knock-in brains, with more cell-cycle activation in homozygotes compared with heterozygotes (Fig. 1E; Supplemental Fig. S2B).

**Figure 2.**
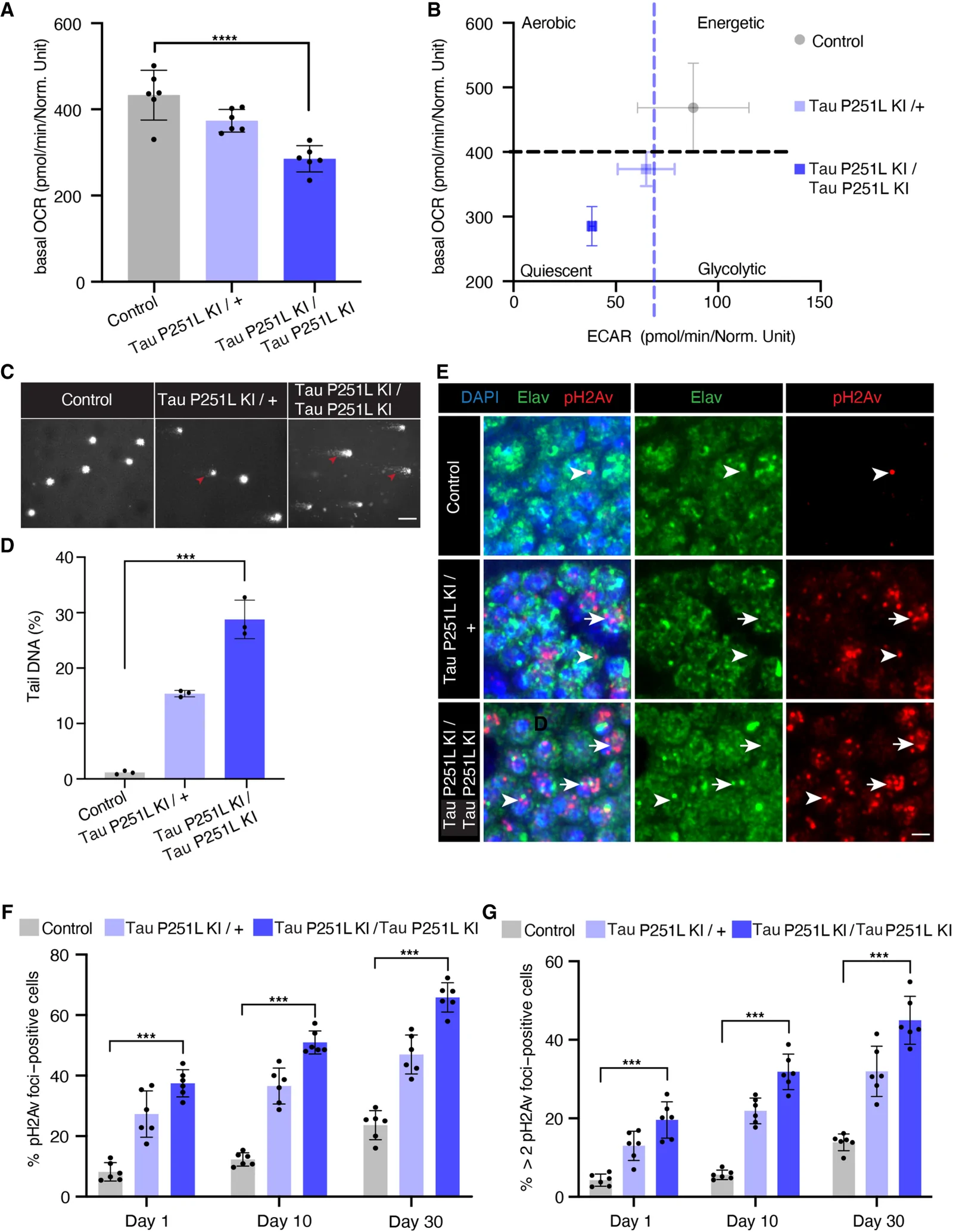
Mitochondrial dysfunction and DNA damage in Tau P251L knock-in brains. (*A*,*B*) Decreased oxygen consumption rate (OCR; *A*) and shift to a quiescent metabolic phenotype as indicated by plotting the OCR versus the extracellular acidification rate (ECAR; *B*) in homozygous and heterozygous Tau P251L knock-in brains compared with controls. n = 6 per genotype. (*C*,*D*) Elevated levels of DNA damage in Tau P251L knock-in brains as indicated by increased tail length (arrowheads) following electrophoresis of nuclei from dissociated brains in the comet assay. n = 3 per genotype. (*E*) Increase in the number of Kenyon cells neurons (identified by the neuronal marker elav) containing DNA double-strand breaks as marked by pH2Av foci (arrowheads; arrows indicate neuronal nuclei with more than two foci) in histological sections of mushroom bodies (Kenyon cells) from Tau P251L knock-in animals, as quantified in *F*,*G*. n = 6 per genotype and time point. Scale bars represent 5 µm. Flies are 10 d old in *A*–*D*, 30 d old in *E*, and the age indicated in the figure labels in *F*,*G*. Data are presented as mean ± SD. (***) *P* < 0.001, one-way ANOVA with Tukey post-hoc analysis.

Metabolic alterations and mitochondrial dysfunction are pervasive features of neurodegenerative diseases, including tauopathies (DuBoff et al. 2013; Götz et al. 2019). We thus performed metabolic analysis on intact whole-fly brains using the Seahorse XFe96 analyzer (Neville et al. 2018). We observed a reduced basal oxygen consumption rate (OCR) and a shift in mitochondrial bioenergetics to quiescent metabolic state in Tau 251L knock-in animals, with homozygotes showing more impairment than heterozygotes (Fig. 2A,B).

Oxidative stress accompanying mitochondrial dysfunction results in damage to key cellular substrates, including DNA. DNA damage commonly occurs in age-related neurodegenerative diseases (Welch and Tsai 2022), including tauopathies (Khurana et al. 2012; Shanbhag et al. 2019; Thadathil et al. 2021). We took two approaches to examining DNA damage in Tau P251L knock-in animals. First, we used the comet assay, in which DNA single- or double-strand breaks are showed using single-cell gel electrophoresis (Khurana et al. 2012; Frost et al. 2014). We observed that nuclei from the brains of Tau P251L knock-in flies displayed almost twofold longer comet tails than those of controls (Fig. 2C, arrowheads, D).

As a second measure of DNA damage, we immunostained for the histone variant H2Av phosphorylated at serine 137 (pH2Av), a marker of DNA double-strand breaks (Madigan et al. 2002; Khurana et al. 2012; Frost et al. 2014). We found significantly increased numbers of double-strand breaks within neurons (Fig. 2E, arrows, arrowheads, F,G). DNA double-strand breaks were elevated with age and in homozygous compared with heterozygous Tau P251L knock-in flies (Fig. 2E–G). Increased DNA damage was assessed by counting both the numbers of Kenyon cell nuclei containing pH2Av foci and the numbers of Kenyon cell nuclei containing more than two foci (Fig. 2E, arrows, G), which correlate with increased numbers of DNA double-strand breaks (Hong and Choi 2013; Lapytsko et al. 2015).

### Single-cell RNA sequencing reveals gene expression changes mediated by pathologic Tau

**Figure 3.**
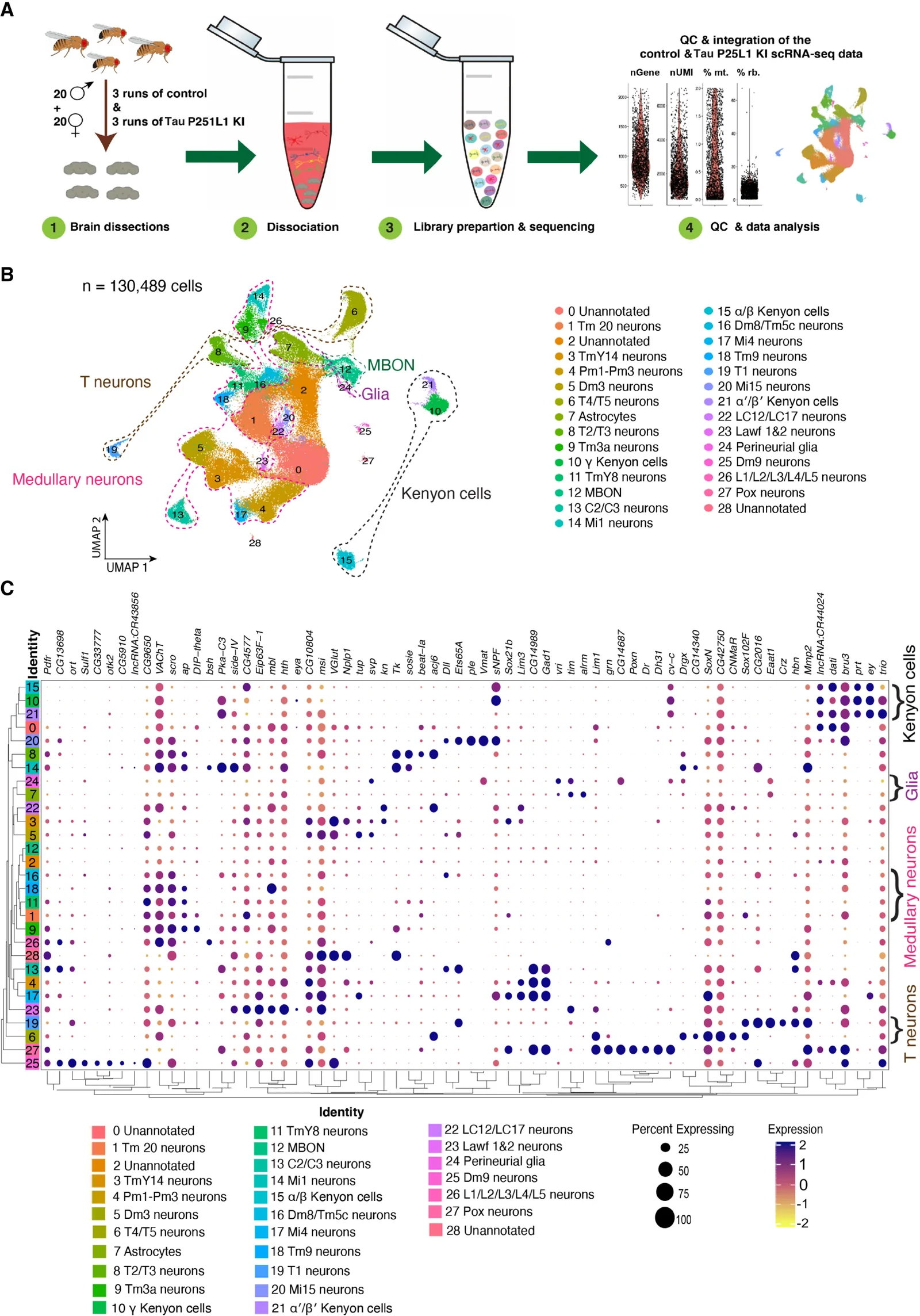
Single-cell RNA sequencing (scRNA-seq) of Tau P251L knock-in brains. (*A*) Schematic of the scRNA-seq analysis pipeline. Following dissection, brains were dissociated in the enzymatic solutions, and the single-cell suspension was encapsulated by the 10x Genomics Chromium platform. The 10x libraries were prepared and sequenced, and after quality control, data were analyzed. (*B*) UMAP representation of the six integrated scRNA-seq runs: three control and three Tau P251L knock-in. The integrated data set contains 130,489 cells, and 26 clusters out of 29 were annotated. (*C*) Percentage expression heatmap of the highly expressed marker genes within all clusters. Flies are 10 d old.

Our Tau P251L knock-in flies replicate important features of human tauopathies and transgenic models of the disorders. We therefore performed single-cell RNA sequencing (scRNA-seq) to investigate transcriptional programs and cellular pathways altered by expression of mutant Tau. Using an optimized brain dissociation method, 10x library preparation, sequencing, and a bioinformatics analysis pipeline, we implemented scRNA-seq on Tau P251L knock-in and control *Drosophila* brains at 10 d of age (Fig. 3A). The 10-d time point was chosen to identify early perturbations related to neuronal dysfunction and degeneration (Figs. 1, 2). After stringent quality control, 130,489 high-quality cells were retained in the final integrated data set, and 29 clusters of cells were identified. We annotated 26 clusters using a published fly cell atlas (Li et al. 2022). We used the most highly expressed marker genes within each cluster to identify clusters. For instance, we used *dac*, *crb*, and *jdp* to annotate Kenyon cells; *Yp1*, *Yp2*, and *Yp3* for mushroom body output neurons (MBONs); *Mtna*, *CG8369*, and *CG1552* for glia; *CG34355*, *Gad1*, and *mamo* for medullary neurons; and *acj6*, *Li1*, and *sosie* for T neurons (Supplemental Figs. S3–S5). The clustered dot plot illustrates enrichment of marker genes in annotated neuronal and glial clusters (Fig. 3C; Supplemental Fig. S4C). Based on prior published analyses (Croset et al. 2018; Davie et al. 2018; Li et al. 2022), we further outlined major groups of cells, including Kenyon cells, medullary neurons, MBONs, astrocytes, and perineurial glia (Fig. 3B; Supplemental Fig. S3; Supplemental Table S1). As previously observed (Davie et al. 2018), cholinergic neurons were the most common neuronal type defined by neurotransmitter phenotype, followed by GABAergic and glutamatergic neurons (Supplemental Fig. S3). Less abundant clusters of dopaminergic neurons were also identified (Supplemental Fig. S3). In summary, our scRNA sequencing in a precisely edited *Drosophila* tauopathy model, yielded 130,489 high-quality cells and identified cellular populations throughout diverse brain regions and cell types, including rarer cell populations such as astrocytes and perineurial glia.

**Figure 4.**
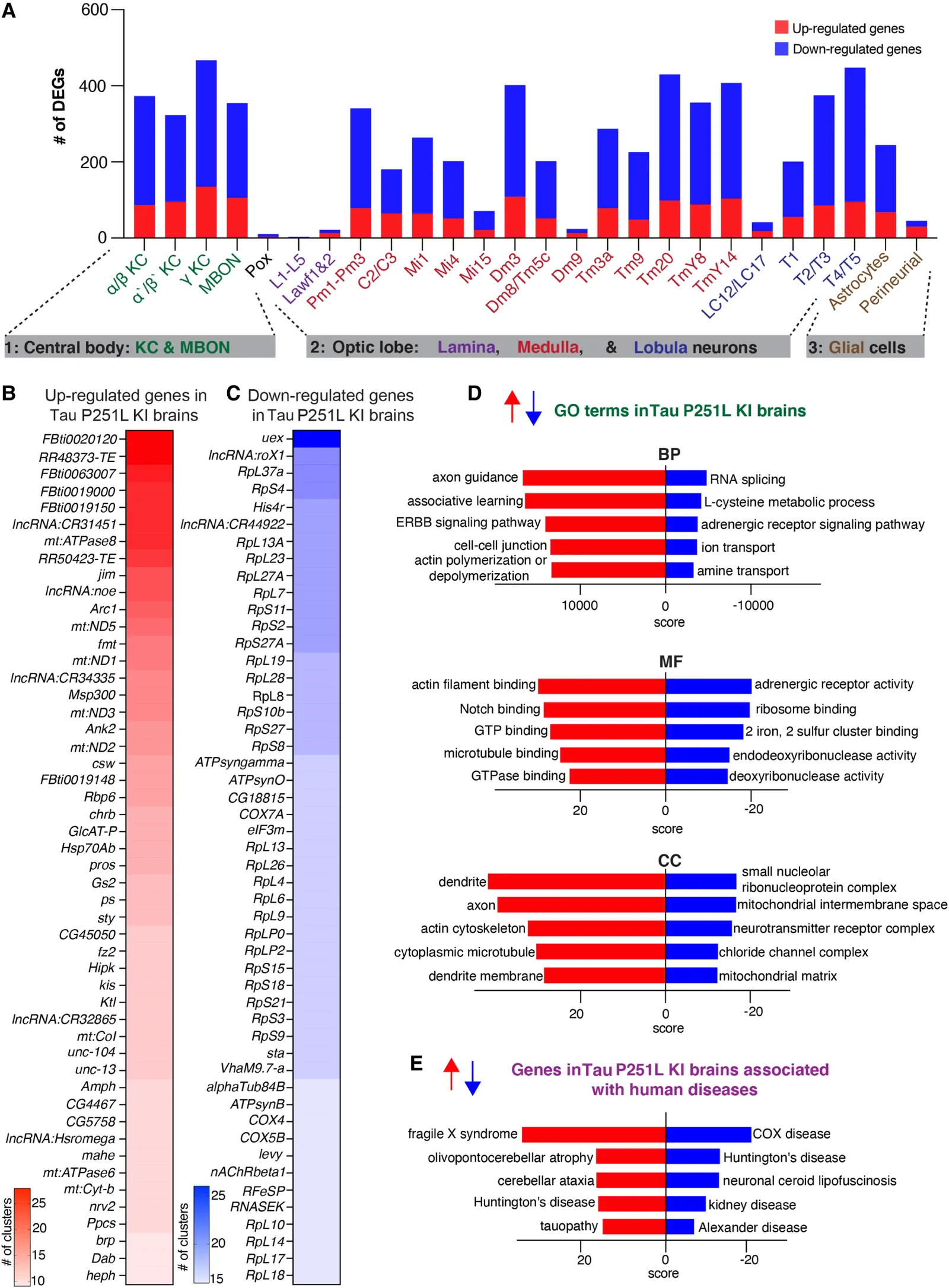
Differential gene expression and enrichment analysis of the scRNA-seq data set in Tau P251L knock-in brains compared with controls. (*A*) The number of differentially expressed genes (DEGs), both up-regulated and down-regulated genes, in all the annotated clusters of Tau P251L knock-in brains compared with controls. Results are displayed across three major anatomic and functional classes of cells: (1) central body containing three clusters of Kenyon cells (KCs), mushroom body output neurons (MBONs) and pox neurons; (2) optic lobe neurons containing lamina, medullary, and lobula neurons clusters; and (3) glia cells containing astrocytes and perineurial clusters. (*B*,*C*) Heatmaps of the top 50 up-regulated (*B*) and down-regulated (*C*) genes in all the clusters of Tau P251L knock-in brains compared with controls (Supplemental Table S3). (*D*) Gene Ontology (GO) enrichment analysis identified top up-regulated and down-regulated biological processes (BPs), molecular functions (MFs), and cellular components (CCs). (*E*) Analysis of human disease–associated genes revealed top up-regulated and down-regulated disease-associated gene sets. Score represents the combined score c = log(p) × z (Chen et al. 2013).

After sample integration, quality control, and cluster annotation, we performed differential gene expression (DEG) analysis to identify genes modulated by precise pathologic mutation modeling of tauopathy in the *Drosophila* brain. DEG analysis of all the 26 annotated clusters revealed that Tau P251L knock-in altered genes throughout the *Drosophila* brain, in both neurons and glia (Fig. 4A; Supplemental Table S2). We found that 472 genes were up-regulated across all clusters in Tau P251L knock-in brains, whereas 1145 genes were down-regulated (Supplemental Table S3). Transposable elements (*FBti0020120 RR48373-transposable-element*, *FBti0063007*, *FBti0019000*, *FBti0019150*, *RR50423-transposable-element*, *FBti0019148*) were frequently up-regulated in P251L knock-in brains (Fig. 4B), consistent with findings from *Drosophila* human *TAU* overexpression models and human Alzheimer's disease brain tissue (Guo et al. 2018; Sun et al. 2018). The set of commonly down-regulated genes was notable for multiple ribosomal protein genes (Fig. 4C), suggesting a translational defect in tauopathy. Multiple nuclear and mitochondrially encoded respiratory chain subunits and other mitochondrial proteins were notably present in the commonly up-regulated and down-regulated genes, as were genes encoding cytoskeletal and associated proteins (*Arc1*, *Msp300*, *Ank2*, *unc-104*, *Amph*, *brp*, *alphaTub84B*). Both categories of genes fit well with known mediators of tauopathy pathogenesis (DuBoff et al. 2013; Schulz et al. 2023).

As expected, gene enrichment analyses (Fig. 4D; Supplemental Fig. S6) highlighted mitochondrial and cytoskeletal processes. In addition, diverse metabolic and neuronal function pathways, including associated learning, previously associated with Alzheimer's disease and related tauopathies emerged from Gene Ontology (GO) enrichment analyses. Enrichment analysis for human disease–associated genes revealed predominantly neurodegenerative disorders, including tauopathy (Fig. 4E).

### Distinct and shared region- and cell-specific transcriptional programs in Tau P251L knock-in brains

Significant anatomic and cell type selectivity characterizes human neurodegenerative diseases, including tauopathies. We therefore analyzed gene expression changes separately in anatomically and functionally related groups of cells, including the central body (Kenyon cells, MBON, and Pox neurons), optic lobe (lamina, medulla, and lobula neurons), and glia (astrocytes and perineurial glia). Volcano plots in Supplemental Figure S7, A, C, and F, present up-regulated and down-regulated genes in each group of cells. Transposable element were the top up-regulated genes in each of the three groups. GO enrichment analyses (Supplemental Fig. S7B,D) identified distinct biological processes altered by mutant *tau* expression in the central body compared with the optic lobe. Both associative learning and cAMP metabolic process were specifically identified in the central body, correlating with the importance of Kenyon cells in learning and memory in flies and with the central role for cAMP underlying learning and memory (Feany and Quinn 1995; Guven-Ozkan and Davis 2014). Heterochromatin organization and DNA repair, both processes strongly implicated in tauopathy pathogenesis (Fig. 2; Khurana et al. 2012; Frost et al. 2014; Welch and Tsai 2022), emerged as enriched processes following analysis of the central body and optic lobe separately (Supplemental Fig. S7D). Direct comparison of differentially regulated genes in the central body compared with the optic lobe neurons revealed 239 commonly regulated genes and 562 distinct genes (Supplemental Fig. S7E). Consistent with analysis of the total transcriptome (Fig. 4), shared biological processes included down-regulation of mitochondrial genes and up-regulation of axon guidance–associated genes (Supplemental Fig. S7E; Supplemental Table S4).

Because *tau* is a predominantly neuronally expressed gene (Heidary and Fortini 2001; Goedert 2004; Götz et al. 2019), the observed changes in neuronal transcriptomes may reflect cell-autonomous effects of the frontotemporal dementia associated mutant TAU protein. Our single-cell approach also revealed significant changes in gene expression in glial cells in Tau P251L knock-in brains (Supplemental Fig. S7F,G). Expression of mutant *tau* may thus exert non-cell-autonomous control on glial transcriptional programs. Metabolic processes (Supplemental Fig. S7G) were down-regulated in glia in response to neuronal expression of mutant *tau*, consistent with the importance of glial metabolism in supporting a wide array of neuronal functions (Nedergaard and Verkhratsky 2012; Verkhratsky et al. 2012). The top two GO processes identified by analysis of up-regulated glial genes were associative learning and regulation of neuronal remodeling, suggesting that coordinate changes in neurons and glia may lead to impairment of critical neuronal functions when mutant *tau* is expressed by neurons.

**Figure 5.**
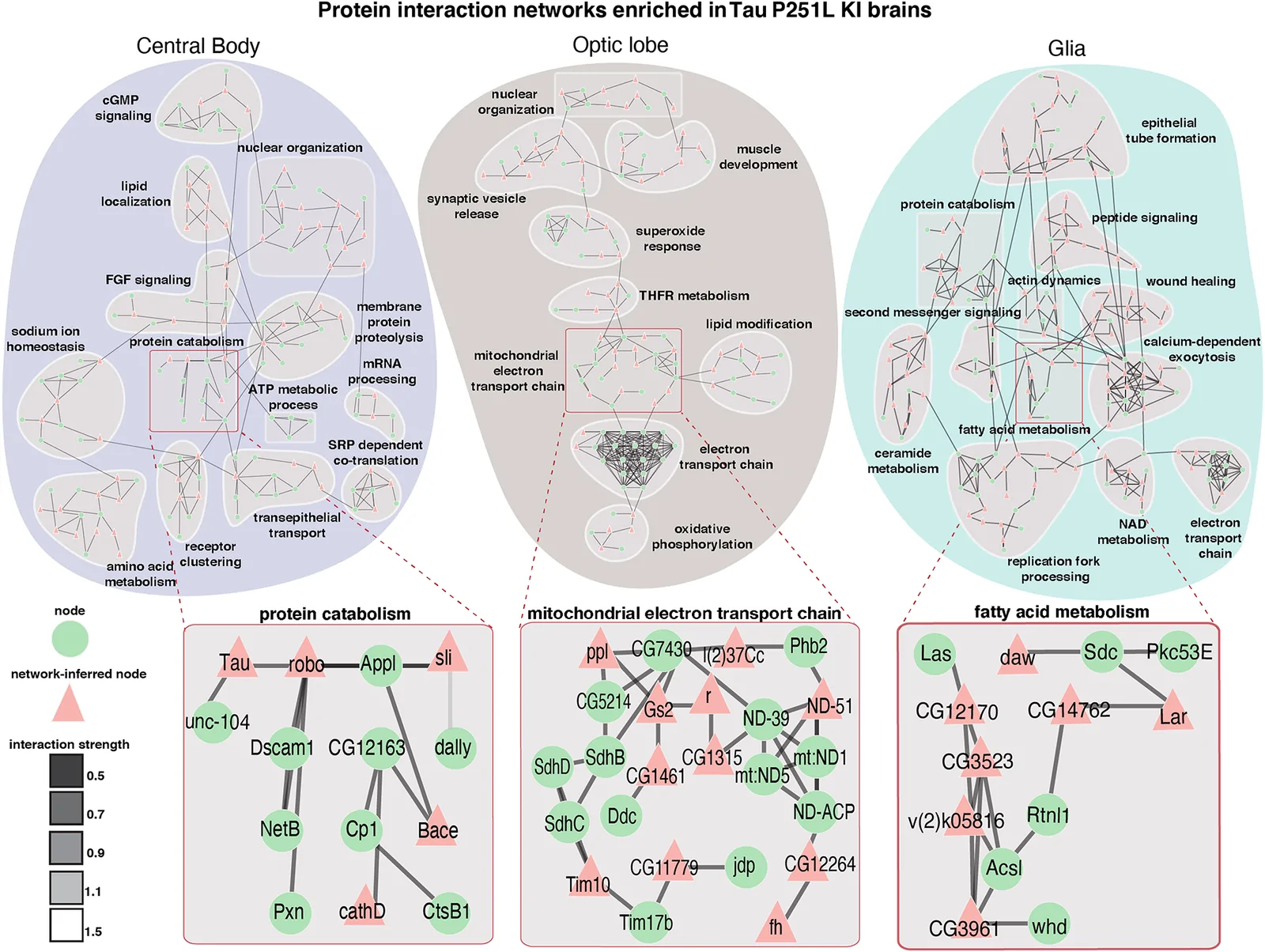
Protein interaction networks enriched in the central body, optic lobe, and glia in Tau P251L knock-in brains compared with controls. Protein interaction networks are largely distinct among central body neurons, optic lobe neurons, and glia. Subnetworks including nodes enriched for protein catabolism (central body), electron transport chain (optic lobe), or fatty acid metabolism (glia) are highlighted. Interaction strength displayed in gray shows the stringency of the interaction: The lower the strength, the stronger the interaction.

We next constructed protein interaction networks to explore further the biological pathways altered in Tau P251L knock-in brains compared with controls. We used the solution of the prize-collecting Steiner forest algorithm (Tuncbag et al. 2013) to map differentially expressed genes onto a network of physical protein interactions using *Drosophila* interactome data. Networks constructed from the central body, optic lobe, and glial cells were substantially distinct (Fig. 5), consistent with differential effects of mutant Tau on different anatomic regions and cell types. The electron transport chain was identified in subnetworks from both the optic lobe and glia, raising the possibility that mutant Tau can influence mitochondrial function in both a cell-autonomous and a non-cell-autonomous fashion (Figs. 2, 5). Regulation of nuclear function was commonly identified in both central body and optic lobe neurons, consistent with a strong influence of neuronally expressed Tau on chromatin structure mediated through the linker of nucleoskeleton and cytoskeleton (LINC) complex (Frost et al. 2014, 2016).

Protein catabolism was a subnetwork in both central body and glial networks. Protein catabolism was connected to multiple other subnetworks in the central body network and contained multiple proteins previously implicated in Alzheimer's disease, including Appl (fly ortholog of APP), beta-site APP-cleaving enzyme (Bace; a fly homolog of BACE1), three members of the cathepsin family (CtsB1, cathD, CtsF/CG12163), and Tau itself identified as a computational network-inferred node. As expected from GO analysis (Supplemental Fig. S7G), multiple metabolic subnetworks were identified in the glial network, consistent with the role of glia in providing metabolic support to neurons (Nedergaard and Verkhratsky 2012; Verkhratsky et al. 2012). A subnetwork enriched for nodes associated with fatty acid metabolism was identified in the glial network (Fig. 5), correlating with the important role of glia in lipid metabolism and signaling in both flies and mammalian systems (Lee et al. 2021; Goodman and Bellen 2022). Detailed protein interaction networks identified in the central body, optic lobe, and glia are shown in Supplemental Figures S8 through S10.

### Cell–cell communication and pseudotime trajectory analyses highlight the role of glial cells in Tau P251L knock-in brains

**Figure 6.**
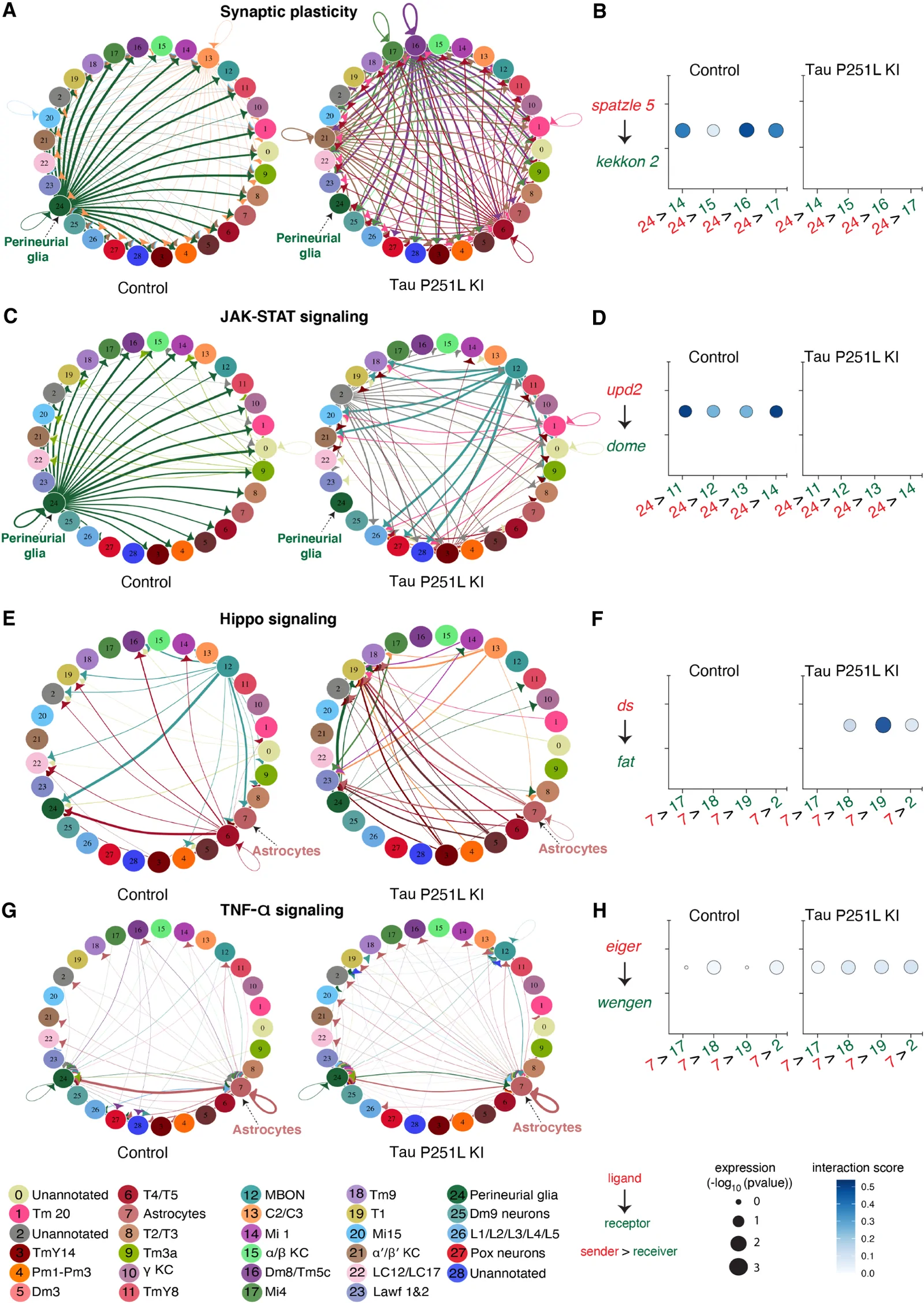
Cell–cell communication analysis predicts altered signaling in Tau P251L knock-in brains compared with controls. (*A*) Altered ligand and receptor expression predicts regulation of synaptic plasticity signaling mainly via perineurial glial cells in control brains. (*B*) Signaling from perineurial glia is significantly reduced in Tau P251L knock-in brains, as predicted by levels of spaetzle ligand and kekkon receptor. (*C*,*D*) JAK-STAT signaling, as predicted by expression of the upd2 ligand and dome receptor, mediated by perineurial glia in control brains (*C*), is substantially reduced in brains from Tau P251L knock-in animals (*D*). (*E*,*F*) Hippo signaling, indicated by expression of ds ligand and fat receptor, is up-regulated in astrocytes of flies expressing P251L mutant Tau compared with controls. (*G*,*H*) Predicted TNF-α signaling from ligand eiger to receptor wengen is increased in astrocytes of Tau P251L knock-in flies. In panels *B*,*D*,*F*,*H*, the interactions from and to the specified cell types are indicated on the *x*-axis, the size of the circle indicates the *P-*value, and the intensity of the blue color illustrates the interaction score as defined in the figure label *below* the panels.

Altered gene expression (Supplemental Fig. S7) and protein interaction networks (Fig. 5) in glia driven by neuronal-predominant expression of P251L mutant Tau suggest perturbed intercellular communication in P251L knock-in brains. We therefore calculated the interaction scores for 196 manually curated ligand–receptor pairs using the FlyPhoneDB quantification algorithm (Liu et al. 2022) in Tau P251L knock-in brains and controls. We found significant alterations predicted in major cellular signaling pathways (Fig. 6; Supplemental Fig. S11). Altered signaling is indicated in circle plots in Figure 6 (A,C,E,G) by nodes representing unique cell types and by edges representing a communication event. The thickness of an edge reflects the interaction strength of the communication event. Dot plots in Figure 6 (B,D,F,H) display the calculated score of selected ligand–receptor pairs from one cell type to another, with the shading of the dot indicating the interaction score and with the size of the dot indicating the the *P-*value. Many of predicated signaling changes support altered communication between glia and neurons. For instance, synaptic plasticity signaling, assessed by expression of the ligand spatzle and kekkon receptors, was mainly driven by perineurial glia in the control brain. However, perineurial glial cells in Tau P251L knock-in animals had reduced expression of the ligand spatzle 5, whereas recipient cells down-regulated kekkon receptors (Fig. 6B). Similarly, expression of the JAK-STAT ligand upd2 was significantly down-regulated in perineurial glia in Tau P251L knock-in brains compared with those of controls, whereas the receptor dome was reduced in expression in widespread target neuronal clusters (Fig. 6D). There was a predicted up-regulation of JAK-STAT signaling from MBONs to a restricted set of neuronal clusters in brains of flies expressing P251L mutant Tau (Fig. 6C). In contrast, predicted hippo signaling from MBONs to perineurial glial based on decreased levels of the ds ligand and receptor fat was decreased in Tau P251L knock-in brains compared with controls (Fig. 6E).

Astrocytic signaling also showed predicted changes in Tau P251L knock-in brains compared with controls. JAK-STAT signaling perineurial glia to astrocytes was reduced in mutant Tau-expressing brains (Fig. 6C), whereas hippo signaling from astrocytes to multiple neuronal subtypes was increased in Tau P251L knock-in brains (Fig. 6E,F). TNF-α signaling from astrocytes was also increased in flies expressing mutant Tau, as suggested by increased levels of the ligand eiger and receptor wengen (Fig. 6G,H). Altered astrocyte integrin, hedgehog, and insulin signaling was also suggested by changes in expression of ligand and cognate receptor pairs (Supplemental Fig. S11A,D,E).

**Figure 7.**
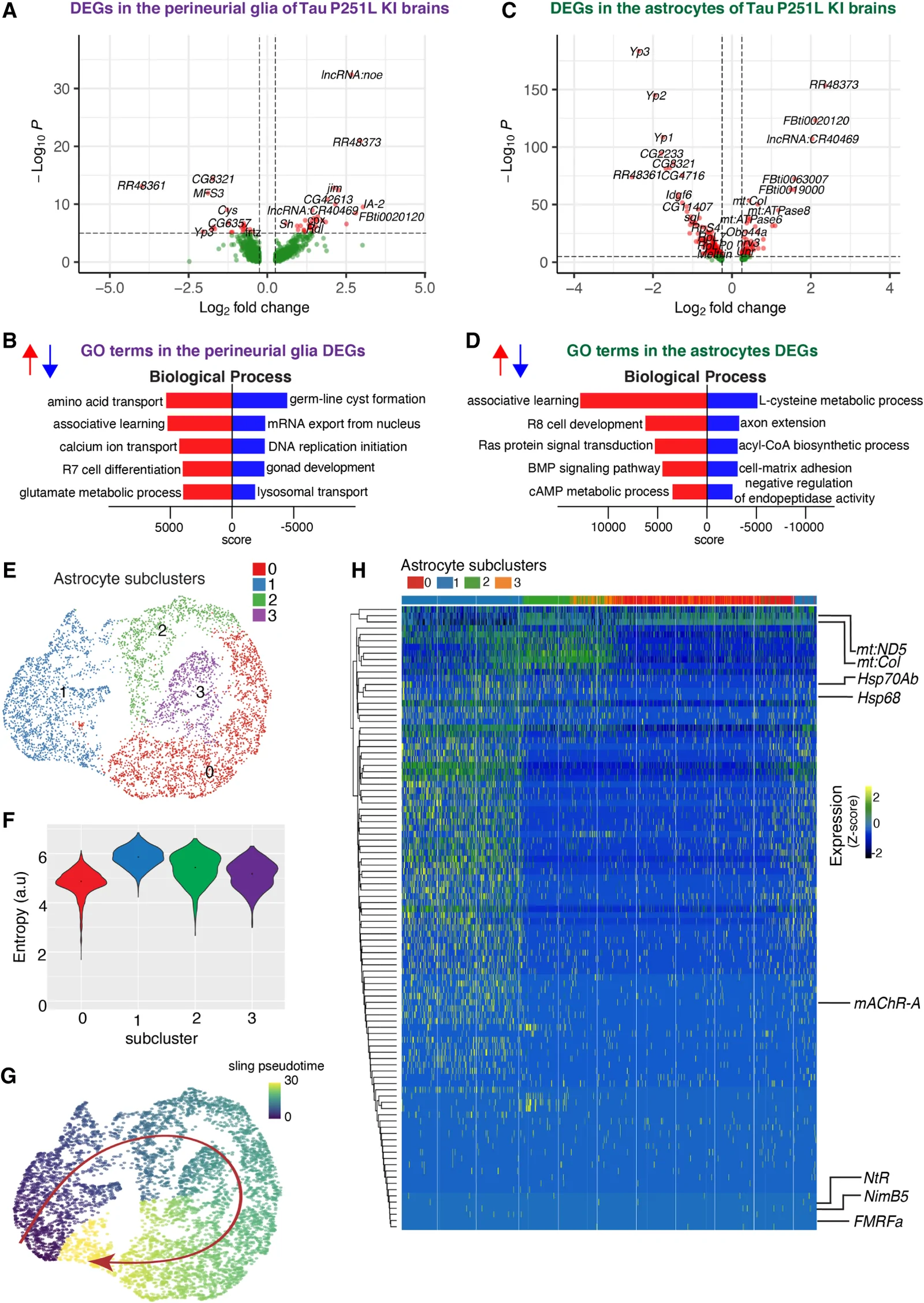
Gene expression and trajectory analysis in glia. (*A*) Differentially regulated genes, both up-regulated and down-regulated, in perineurial glia of Tau P251L knock-in brains compared with controls. (*B*) GO analysis shows biological processes associated with the up-regulated and down-regulated genes in perineurial glia from Tau P251L knock-in brains compared with controls. (*C*) Differentially regulated genes, both up-regulated and down-regulated, in astrocytes of Tau P251L knock-in brains compared with controls. (*D*) GO analysis shows biological process associated with up-regulated and down-regulated genes in astrocytes of Tau P251L knock-in brains. All dots on the volcano plots are significant at FDR < 0.05 and log_2_FC > 0.25 for up-regulated and log_2_FC< −0.25 for down-regulated genes. Score represents the combined score c = log(p) × z (Chen et al. 2013). Astrocytes from both control and Tau P251L knock-in brains were further subclustered into four groups. (*E*,*F*) Entropy analysis to define the root for trajectory analysis revealed cluster 1 to have the highest entropy. (*G*) Slingshot trajectory analysis on astrocyte clusters identified a single lineage passing sequentially from clusters 1 to 2, 3, and 0. (*H*) Differential gene expression between astrocyte subclusters adjacent in pseudotime were used to cluster genes along the pseudotime trajectory. Each row in the heat map represents a gene. The columns are astrocyte subclusters arranged according to pseudotime from *left* to *right*. Examples of differentially regulated genes from enriched GO biological processes are shown on the calculated trajectory.

Given the altered gene expression (Fig. 4; Supplemental Fig. S7), protein interaction networks (Fig. 5), and predicted signaling (Fig. 6) in glia, we next examined gene expression profiles in these nonneuronal cells in more detail (Fig. 7). Transposable elements were significantly up-regulated in both types of glia (Fig. 7A,C; Supplemental Table S5), although one transposable element was highly down-regulated in both glia subsets (*RR48361*). GO enrichment analysis highlighted different metabolic pathways in the two cell types. Amino acid and glutamate metabolism pathways were enriched in perineurial glia, whereas L-cysteine, acyl-CoA, and cAMP metabolic pathways were enriched in astrocytes (Fig 7B,D).

Because we observed significant alterations in glial signaling in Tau P251L knock-in brains (Fig. 6; Supplemental Fig. S11), we investigated glial gene trajectories in our scRNA-seq, focusing on astrocytes because we obtained a large number (nearly 5800) of these cells (Supplemental Table S1). We first subclustered astrocytes into four groups (Fig. 7E). We then calculated the entropy of these clusters (Guo et al. 2017) and used cluster 1, which showed the highest entropy (Fig. 7F), as the root for trajectory analysis (Street et al. 2018). A single lineage starting from cluster 1 and progressing sequentially from cluster 2 through cluster 3 and finally to cluster 0 emerged (Fig. 7G). We then clustered differentially expressed genes along the calculated trajectory as presented in the heat map, in which pseudotime is represented in columns from left to right (Fig. 7H). Our pseudotemporal analysis suggests different stages of astrocytic response to tauopathy.

GO analysis across pseudotime revealed multiple genes involved in signaling pathways (*FMRFa*, *NimB5*), particularly in cholinergic signaling (nicotinic acetylcholine receptor subunit *NtR*, *mAChR-A*, *ChAT*) early in the glial trajectory. Cellular stress response emerged later in the trajectory with up-regulation of heat shock proteins (*Hsp68*, *Hsp70Ab*), whereas altered mitochondrial gene expression (*mt:ND5*, *mt:Col*) characterized astrocytes late in the calculated trajectory. These findings suggest that altered astrocyte signaling (Fig. 6; Supplemental Fig. S11) may emerge early in tauopathy pathogenesis and drive subsequent cell-autonomous and non-cell-autonomous stress responses and cytotoxicity. A complete list of all differentially expressed glial genes, genes associated with GO biological processes, and trajectory-associated genes is provided in Supplemental Table S5.

### Gene regulatory networks in control and Tau P251L knock-in Kenyon cells

**Figure 8.**
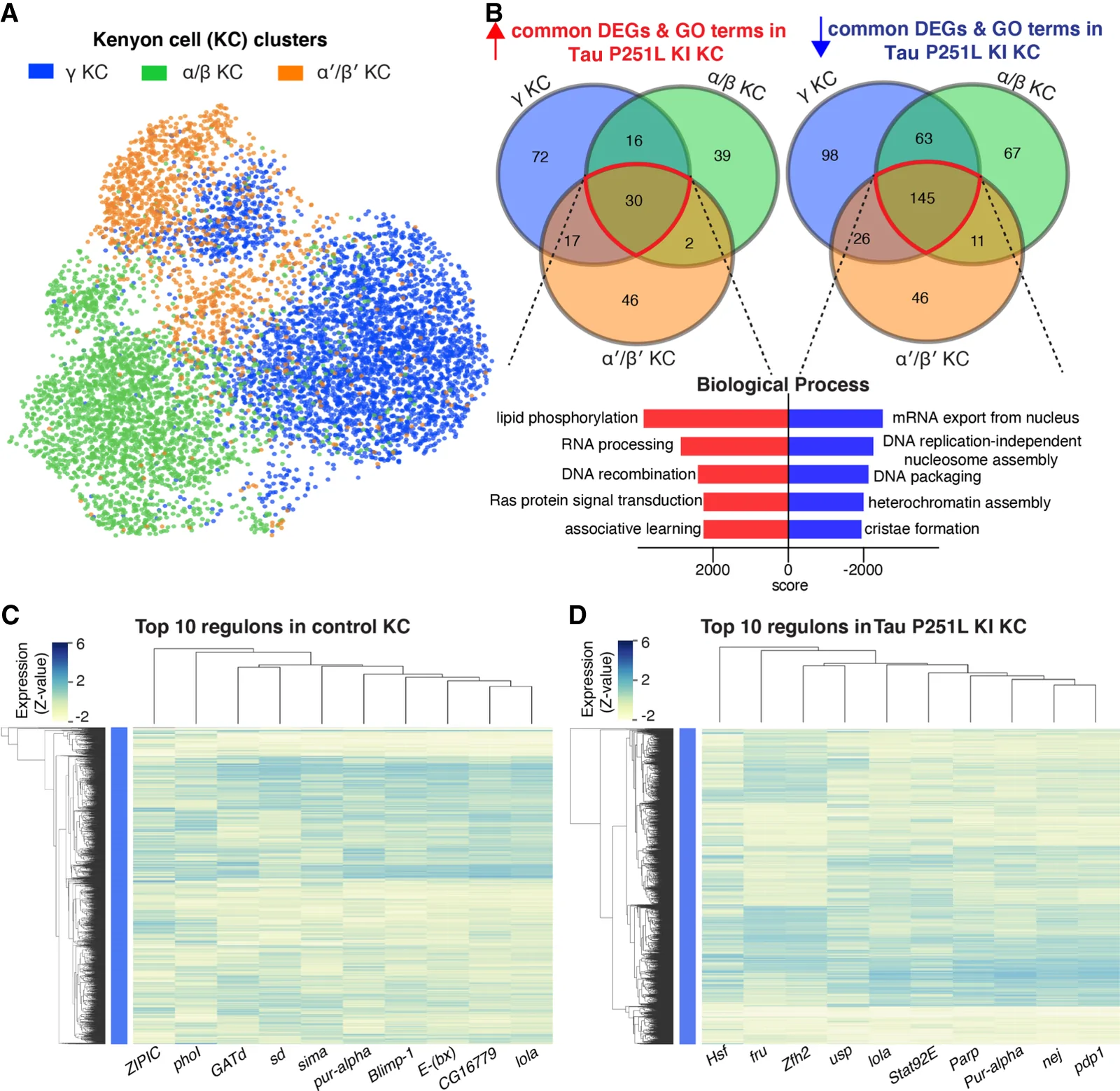
Gene expression and regulatory networks in Kenyon cells (KCs). (*A*,*B*) Three KC clusters—gamma-KC, alpha/beta-KC, and alpha′/beta′-KC—and biological process based on the common up-regulated and down-regulated genes in KC clusters in Tau P251L knock-in brains. Score represents the combined score c = log(p) × z (Chen et al. 2013). Control and Tau P251L knock-in Kenyon cells were clustered separately using SCENIC gene regulatory network analysis to identify regulons. (*C*,*D*) The top 10 regulons identified by SCENIC gene regulatory network analysis in control (*C*) and Tau P251L knock-in (*D*) KCs are presented in the heatmaps. Each row represents a KC; each column is a regulon.

Kenyon cells are a major defined neuronal component of the central body of the *Drosophila* brain (Fig. 4). Together with their output neurons (MBONs), Kenyon cells play a central role in learning and memory in the *Drosophila* brain (Heisenberg 2003; Modi et al. 2020); memory loss is a key feature of human tauopathies (Grossman et al. 2023). Kenyon cells are cholinergic (Barnstedt et al. 2016), a neuronal type that is selectively vulnerable in previously described fly tauopathy models (Wittmann et al. 2001), and a pathway altered early in our trajectory analysis (Fig. 7). Our cell–cell communication analyses suggested altered signaling in Kenyon cells, or their output neurons, via multiple signaling pathways (Fig. 6; Supplemental Fig. S11). We therefore focused next on gene expression in Kenyon cells. We identified three Kenyon cells clusters: gamma-Kenyon cells, alpha/beta-Kenyon cells, and alpha′/beta′-Kenyon cells (Fig. 8A). Transposable elements were up-regulated in all Kenyon cell clusters in Tau P251L knock-in brains (Supplemental Fig. S12A,C,E), as observed in other neuronal and glial clusters (Fig. 7; Supplemental Fig. S7). Analysis of biological pathways associated with common up-regulated and down-regulated genes in all three Kenyon cell clusters identified key biological processes previously linked to tauopathy pathogenesis (Frost et al. 2015; Götz et al. 2019), including control of DNA and RNA structure and metabolism (Fig. 8B), as well as many pathways without prior links to tauopathy. A complete list of differentially expressed genes and associated biological processes is given in Supplemental Table S6.

Given the multiple lines of evidence connecting tauopathy pathogenesis to Kenyon cell function, we next determined the gene regulatory networks controlling disease-associated changes in gene expression in Kenyon cells. We implemented the single-cell regulatory network inference and clustering (SCENIC) (Aibar et al. 2017) workflow on gene expression data from control and Tau P251L knock-in Kenyon cells. The top 10 regulons identified in control cells compared with tauopathy model Kenyon cells are show in columns in the heat maps in Figure 8, C (control Kenyon cells) and D (Tau P251L knock-in Kenyon cells). Regulons were largely distinct in the two genotypes (Fig. 8C,D; Supplemental Table S7). The shared transcription factors among the top 10 regulons were *lola* and *pur-alpha*. Even for the shared regulons, the gene expression patterns per cell clustered and coexpressed with different transcription factors and are different among Kenyon cells of control versus Tau P251L knock-in animals. The distinct gene regulatory networks illustrated in the heatmap are concordant with altered gene expression (Fig. 8B) and cell–cell communication (Fig. 6) between control and Tau P251L knock-in Kenyon cells. The increase in *HSF*, *Stat92E*, and *Parp* expression (Supplemental Fig. S13) and regulons (Fig. 8D) in brains of tauopathy model flies are consistent with elevated cellular stress, DNA damage, and cell death in aging neurons exposed to mutant Tau P251L (Figs. 1, 2).

## Discussion

Here we present a new model of tauopathy in the experimentally facile model organism *Drosophila* based on precise gene editing of the endogenous *tau* gene to introduce a mutation orthologous to human proline 301 to leucine (P301L), the most common *TAU* mutation in frontotemporal dementia patients (Poorkaj et al. 2001). We observe age-dependent neurodegeneration in our knock-in animals (Fig. 1C,D). Homozygous knock-in flies display early and greater total levels of degeneration compared with heterozygous animals. These findings are compatible with a toxic gain-of-function mechanism, as generally posited in familial frontotemporal tauopathies (Goedert et al. 2012; Frost et al. 2015; Bardai et al. 2018b; Götz et al. 2019). However, given the important role of microtubules in neurodevelopment, a loss-of-function component contribution cannot be excluded, even given the lack of clear neurodegeneration in *Tau* knockout mice (Harada et al. 1994; Dawson et al. 2001; Morris et al. 2013) and flies (Burnouf et al. 2016). As expected given that levels of mutant Tau are controlled by the endogenous *tau* promotor in our model compared with the strong exogenous promotor systems used in prior transgenic models, neurodegeneration in knock-in animals is observed at older ages and is milder (Wittmann et al. 2001; Bardai et al. 2018b; Law et al. 2022). However, we do observe key biochemical and cellular pathologies previously described in transgenic *Drosophila* tauopathy models, including metabolic dysfunction (Fig. 2A,B), elevated levels of DNA damage (Fig. 2C–G), and abnormal cell-cycle activation (Fig. 1E; Khurana et al. 2006; 2012; DuBoff et al. 2012; Bardai et al. 2018a). Importantly, these pathways are also perturbed in mouse tauopathy models and tauopathy patients (Herrup and Arendt 2002; Andorfer et al. 2005; Khurana et al. 2012; DuBoff et al. 2013; Frost et al. 2015; Götz et al. 2019; Welch and Tsai 2022).

The similarities of our knock-in model to human tauopathies and prior overexpression tauopathy models, recapitulated in a more faithful genetic knock-in context, motivated us to perform a comprehensive transcriptional analysis in our Tau P251L knock-in brains using scRNA-seq. We recovered a large number (130,489) of high-quality cells, which allowed us to identify the majority of previously annotated neuronal and glial groups from prior single-cell sequencing analyses in the adult fly brain (Davie et al. 2018; Li et al. 2022). Comparing gene expression profiles between control and Tau P251L knock-in animals revealed pervasive dysregulation of genes in neuronal (Figs. 4,8) and glial (Fig. 7) subtypes and throughout different anatomic regions (Fig. 4, Supplemental Fig. S6). These findings are consistent with prior single-cell sequencing studies in flies overexpressing mutant human *TAU* (Praschberger et al. 2023; Wu et al. 2023). We observed regulation of both common and distinct biological pathways when comparing differentially expressed genes across cell subtypes. Transposable elements were notably up-regulated in the complete gene expression set, as well as in specific anatomic regions and neuronal subtypes. These findings correlate with a previously described functional role for transposable element mobilization in *Drosophila* models of tauopathy and in tauopathy patients (Guo et al. 2018; Sun et al. 2018). Mitochondrial function has been strongly linked to neurotoxicity in tauopathies (DuBoff et al. 2013; Frost et al. 2015; Götz et al. 2019) and is a feature of our current model (Fig. 2). We accordingly observed altered expression of mitochondrial genes and biological processes in the complete expression data set (Fig. 4), as well as in separate analyses of the central body, optic lobe (Supplemental Fig. S7), and Kenyon cells (Fig. 8; Supplemental Fig. S12). More importantly, we observed significant alterations in multiple metabolic, cellular communication, and biological pathways not previously implicated in tauopathy pathogenesis (Figs. 4–6), which can now be assessed in tauopathy models and patients for mechanistic relevance and ultimately therapeutic targeting.

Cell type selectivity is a fundamental, and poorly understood, feature of human neurodegenerative diseases, including tauopathies. Our protein interaction networks highlighted regionally specific biology with predominantly distinct nodes appearing in the central body compared with the optic lobe (Fig. 5). Comparative analysis of genes differentially expressed in the central body compared with the optic lobe is consistent with substantial regional differences in the response to mutant *tau* expression with substantially greater numbers of unique compared with common genes up-regulated in the central body versus the optic lobe (Supplemental Fig. S7E). Even within subgroups of Kenyon cells, there are equivalent numbers or more uniquely up- or down-regulated genes compared with commonly regulated genes (Fig. 8B). Our data set thus highlights a substantial set of genes that may contribute to selective neuronal susceptibility in neurodegeneration, including many differentially regulated genes and processes not previously linked to TAU pathobiology.

Although TAU is a predominantly neuronal protein (Heidary and Fortini 2001; Goedert 2004; Götz et al. 2019), we observed significant alteration of glial gene expression in Tau P251L knock-in brains compared with controls (Figs. 4, 7), suggestive of non-cell-autonomous control of glia cell function by neuronally expressed Tau. GO (Fig. 7A,B) and protein interaction network (Fig. 5) analyses highlighted a number of metabolic processes altered in glia by expression of toxic Tau in neurons, including glutamate, lipid, and amino acid metabolism (Figs. 5, 7). Glial uptake and detoxification of neurotransmitters and their metabolites, as well as toxic lipid species, maintain neuronal function and viability. Lipid metabolism is further central to energy production by glial cells, which support highly energy consuming neurons with active synaptic transmission (Smolič et al. 2022; Jiwaji and Hardingham 2023). In addition to glial processes previously implicating in controlling neuronal health, our transcriptional analysis revealed new metabolic and signaling pathways in glia regulated by the expression of mutant Tau (Fig. 7A–C), which can now be explored as non-cell-autonomous mechanisms regulating neuronal function and viability in tauopathy.

An effect of mutant *tau* expression enriched in neurons on glial gene expression implies signaling, and possibly perturbed signaling, between the two cell types. Examination of the expression of 196 ligand–receptor pairs (Liu et al. 2022) indeed supported broad alterations in glial–neuronal communication in Tau P251L knock-in flies (Fig. 6; Supplemental Fig. S11), with mutant *tau* expression perturbing synaptic plasticity, JAK-STAT, hippo, TNF-α, integrin, and EGFR signaling between perineurial cells, astrocytes, and multiple neuronal subtypes. Although prior studies have implicated glial signaling, for example, the JAK-STAT pathway (Colodner and Feany 2010), in non-cell-autonomous control of neurotoxicity, the pervasive nature of the altered signaling suggested by our single-cell transcriptional analyses is unexpected and provides multiple targets for functional testing. Our findings further suggest that a systematic and broad perturbation of intercellular signaling is present in tauopathy, which may require manipulation of multiple pathways to correct and system-level analysis to monitor.

Trajectory analysis has been widely used to order temporal events along developmental pathways but has less often been applied to neurodegenerative disease progression (Fitz et al. 2021; Karademir et al. 2022; Wang et al. 2022; Dai et al. 2023). Given the evidence for altered glial–neuronal communication in our Tau knock-in model, we assessed possible trajectories in the four distinct subgroups of astrocytic glial cells that we defined. Using the astrocyte cluster with the highest entropy as the root (Guo et al. 2017), we identified a single astrocyte trajectory (Fig. 7G). DEG and GO analyses across the trajectory revealed altered expression of neurotransmitter and cell signaling genes first, followed by altered cell stress responses, and finally mitochondrial changes (Fig. 7H; Supplemental Table S5). A number of genes involved in cholinergic signaling were changed early in the glial trajectory. We have previously shown that cholinergic terminals are preferentially vulnerable and degenerate early in a tauopathy model based on transgenic human *TAU* expression in flies (Wittmann et al. 2001). Our trajectory analysis may thus help identify early events in glial-mediated neurodegeneration, including pathways not previously associated with tauopathy (Supplemental Table S5). Glial pathways contributing to neurodegeneration are increasingly recognized as attractive and understudied avenues for therapeutic intervention (Jiwaji and Hardingham 2023). Identifying and intervening in early glial–neuronal signaling events may prevent later, and possibly irreversible, neuronal damage.

Reversing pathological neuronal cell-autonomous programs may provide an alternative or additional method of preventing neuronal dysfunction and death in tauopathies. We focused on Kenyon cells as a group of neurons involved in the behaviorally relevant process of memory and composed of cholinergic neurons, a vulnerable cell type in *Drosophila* (Wittmann et al. 2001) and human (Whitehouse et al. 1981; Ishida et al. 2015) tauopathies, to define transcriptional programs driving neurodegeneration in response to mutant Tau expression. As expected by the multiple neuropathological and cell biological abnormalities present in our knock-in model flies (Figs. 1, 2), we observed substantially distinct regulons in Tau P251L knock-in Kenyon cells compared with controls (Fig. 8C,D). We identified regulons involved in stress responses (*Hsf*, *Stat92E*), including the DNA damage response (*Parp*), as would be expected from the presence of elevated DNA damage in Kenyon cells in our knock-in flies (Fig. 2E–G). We recovered *nej*, the fly ortholog of vertebrate CREB binding protein (also known as CBP), as a top regulon induced in knock-in flies. Increasing levels of nej/CBP are beneficial in fly (Cutler et al. 2015) and vertebrate (Caccamo et al. 2010) models relevant to Alzheimer's disease, suggesting that up-regulation of nej may represent a protective response in Kenyon cells. We also identified multiple regulons not previously associated with neurodegenerative tauopathies (Fig. 8C,D). Therapeutic manipulation of these programs or key transcriptionally regulated mediators will be attractive candidates for evaluation in patient tissue, patient-derived cellular models, and vertebrate models of tauopathy.

The mechanisms transducing the effects of mutant Tau on gene expression are likely multiple and, as yet, only partially characterized. We have previously defined a cascade in which cytosolic Tau binds and stabilizes F-actin (Fulga et al. 2007), leading to signal transduction through the LINC complex, nuclear lamin disruption (Frost et al. 2016), and consequent chromatin relaxation (Frost et al. 2014), promoting aberrant transposable element activation and neurodegeneration (Sun et al. 2018). Other cytosolic targets of Tau may promote transcriptional regulation through parallel mechanisms. For example, Tau-mediated actin hyperstabilization promotes mitochondrial dysfunction and excess production of oxidative free radicals by interfering with mitochondrial dynamics (DuBoff et al. 2012). Oxidative stress may directly contribute to elevated DNA damage in tauopathy (DuBoff et al. 2013; Frost et al. 2016; Bardai et al. 2018b; Götz et al. 2019). However, although TAU is best known as a cytosolic protein involved in regulation of the cytoskeleton, a number of studies have shown that TAU can also be detected in the nucleus (Loomis et al. 1990; Thurston et al. 1996; Cross et al. 2000), where the protein binds DNA (Hua et al. 2003; Sjöberg et al. 2006; Wei et al. 2008; Bukar Maina et al. 2016). Thus, TAU may play a direct role in instructing the nuclear transcriptional programs we have defined (Fig. 8C,D).

In summary, here we develop a genetically precise model of frontotemporal dementia caused by the most common *TAU* mutation found in patients and present a comprehensive picture of gene expression changes and derived protein interaction, cell signaling, and transcriptional networks. We recapitulate neurodegeneration, metabolic dysfunction, and DNA damage, common features of human tauopathies (Goedert 2004; Götz et al. 2019; Welch and Tsai 2022), and confirm that cellular pathways perturbed in overexpression tauopathy models are also dysregulated in the more faithful genetic knock-in context. More importantly, our work suggests previously unsuspected, pervasive alterations in glial–neuronal signaling in tauopathy pathogenesis, implicates many new genes and pathways, and provides a genetic model system in which to test the new hypotheses our data suggest.

## Methods

### Genetics and CRISPR-Cas9 editing

The *Drosophila tau* gene is located on the third chromosome. The guide RNAs targeting the *tau* gene to mutate proline 251 to leucine were identified using Harvard Medical School's DRSC/TRiP “find CRISPRs” tool. The gRNA 5′-CCGGGAGGCGGGGACAAGAAGAT-3′ was cloned into the pCDF3.1 plasmid and injected into the embryos of the TH_attP40 nos-Cas9 strain along with a single-stranded oligo nucleotide donor. The single-stranded oligo nucleotide donor was 150 bp in length and contained a C-to-T transition that resulted in alteration of the codon CCG (proline) to CTG (leucine). Embryos were injected (BestGene) and founder flies obtained. Founder flies were then balanced to obtain homozygous knock-in animals. The mutation was confirmed by PCR. The genotype of knock-in animals in most experiments (Figs. 1, 2C–E, 4–8) was *elav-GAL4/+; tau-P251L knock-in* (homozygous or heterozygous for *tau-P251L knock-in* as specified in figures and legends). In these experiments control animals were *elav-GAL4/+.* In Figure 2, A and B, the genotype of knock-in flies was *w^1118^; tau-P251L knock-in*/*tau-P251L knock-in* (homozygous) or *w^1118^; tau-P251L knock-in*/ *+* (heterozygous) as specified in the figure. In Figure 2, A and B, the genotype of control flies was *w*^*1118*^. The *elav-GAL4* line was obtained from the Bloomington *Drosophila* Stock Center. Patrik Verstreken kindly provided *tau* knockout flies. All crosses and aging were performed at 25°C.

### Assessment of neurodegeneration and metabolism

For sectioning, adult flies were fixed in formalin at 1, 10, and 30 d of age and embedded in paraffin. Vacuole, PCNA, and pH2Av levels were examined using previously described methodology (Fulga et al. 2007; Frost et al. 2014) with additional details provided in the Supplemental Methods. Primary antibodies used include pH2Av (Rockland 600-401-914, 1:100), elav (DSHB 9F8A9, 1:5), GAPDH (Thermo Fisher Scientific MA5-15738, 1:1000), and PCNA (DAKO MO879, 1:500). A polyclonal antibody to *Drosophila* Tau was prepared in rabbits immunized with full-length recombinant Tau protein (Thermo Fisher Scientific) and was used at 1:5,000,000 for western blotting. For all histological analyses, at least six brains were analyzed per genotype and time point. The comet assay and assessment of bioenergetics were performed as previously described (Frost et al. 2014; Sarkar et al. 2020) with additional details provided in the Supplemental Methods. The sample size (n), mean, and SEM are given in the figure legends. All statistical analyses were performed using GraphPad Prism 5.0. For comparisons across more than two groups, one-way ANOVA with Tukey post-hoc analysis was used. For comparison of two groups, Student's *t*-tests were performed.

### scRNA-seq and downstream analyses

A standard sample preparation (Li et al. 2017; Davie et al. 2018), raw data processing (Satija et al. 2015), and downstream analyses such as cell cluster annotation (Hu et al. 2021) GO analysis (Kuleshov et al. 2016), protein–protein interaction network analysis (Tuncbag et al. 2016), cell–cell communication analysis (Liu et al. 2022), trajectory analysis (Street et al. 2018), and gene regulatory network analysis(Van de Sande et al. 2020) were performed as previously described. Detailed methods are presented in the Supplemental Methods.

## Data access

All raw and processed sequencing data generated in this study have been submitted to the NCBI Gene Expression Omnibus (GEO; https://www.ncbi.nlm.nih.gov/geo/) under accession number GSE223345. R code (R Core Team 2016; Butler et al. 2018) that was used to perform Seurat-based integration, trajectory, cell–cell interaction, and PPI network analyses is available at GitHub (https://github.com/bwh-bioinformatics-hub/Single-cell-RNA-seq-of-the-CRISPR-engineered-endogenous-tauopathy-model) and as Supplemental Code. The data, code, protocols, and key lab materials used and generated in this study are listed in a Key Resource Table alongside their persistent identifiers at Zenodo (https://zenodo.org/records/22237380).

## Supplementary Material

Supplement 1

Supplement 2

Supplement 3

Supplement 4

Supplement 5

Supplement 6

Supplement 7

Supplement 8

Supplement 9
