# Supplementary material for: Transcriptional programs mediating neuronal toxicity and altered glial–neuronal signaling in a Drosophila knock-in tauopathy model
Source: Genome Res. 2024 Apr;34(4):590–605. doi: 10.1101/gr.278576.123 (PMC11146598; doi:10.1101/gr.278576.123)
Supplement: Supplement 1 [file Supplemental_Material.pdf]

Supplemental Material

Transcriptional programs mediating neuronal toxicity and altered glial-neuronal signaling in a *Drosophila* knock-in tauopathy model

Hassan Bukhari,[1,2] Vanitha Nithianandam,[1,2] Rachel A. Battaglia,[1,2] Anthony Cicalo,[2,3,4] Souvarish Sarkar,[1] Aram Comjean,[5] Yanhui Hu,[5] Matthew J. Leventhal,[6,7] Xianjun Dong,[2,3,4] and Mel B. Feany[1,2*]

[1]Department of Pathology, Brigham and Women's Hospital, Harvard Medical School, Boston, Massachusetts 02115

[2] Aligning Science Across Parkinson's (ASAP) Collaborative Research Network, Chevy Chase, MD 20815

[3]Genomics and Bioinformatics Hub, Brigham and Women's Hospital, Boston, MA 02115

[4]Department of Neurology, Brigham and Women's Hospital, Harvard Medical School, Boston, MA, 02115

[5]Department of Genetics, Blavatnik Institute, Harvard Medical School, Boston, MA 02115

[6]Department of Biological Engineering, Massachusetts Institute of Technology, Cambridge, MA 02139

[7]MIT Ph.D. Program in Computational and Systems Biology, Cambridge, MA 02139

*Correspondence should be addressed to:

Mel B. Feany, M.D., Ph.D., mel_feany@hms.harvard.edu (tel. (617) 525-4405)

Table of Contents

Supplemental Methods:

Sectioning, immunostaining, and imaging 5

Comet assay ………………………………………………………………………6

Measurement of oxygen consumption and extracellular acidification rates 6

scRNA sequencing: sample preparation 6

Single-cell encapsulation, sequencing, and downstream processing……….7

10x raw data processing …………………..……………………………………7

Seurat data processing 8

Cell cluster annotation, gene enrichment, and Ontology analyses 9

Protein-protein interaction network and cell-cell communication analysis 10

Trajectory analysis 10

Gene regulatory network analyses 11

Supplemental Tables

Supplemental_Table_S1: Top 10 markers of the identified clusters, number of cells, and types of cells in the final integrated dataset **.......................................12**

Supplemental_Table_S2: Normalized read counts of the identified genes in all the clusters **...12**

Supplemental_Table_S3: Differentially regulated genes (DEGs) and Gene Ontology terms in the tau P251L KI brain 12

Supplemental_Table_S4: Up and down-regulated genes and Gene Ontology terms in the major cluster types in the tau P251L KI brain 12

Supplemental_Table_S5: Up and down-regulated DEGs and Gene Ontology in glial cell clusters and pseudotime DEGs in astrocyte clusters 12

Supplemental_Table_S6: Up and down-regulated DEGs and Gene Ontology in the Kenyon cell clusters of tau P251L KI brains compared to controls ……..….12

Supplemental_Table_S7: Details of Regulon identified, via gene regulatory network analysis, in control and tau P251L KI Kenyon cells………..…………13

Supplemental Figures

Supplemental_Fig_S1: Multiple sequence alignment of tau across species ……………………………………………………………………………………14-15

Supplemental_Fig_S2: *Drosophila* tau levels and PCNA staining in tau P251L KI brains compared to controls …………………………………………………………..16-17

Supplemental_Fig_S3: Relative abundance of cells and neuronal identities within the final integrated dataset ……………………………………………………………18-19

Supplemental_Fig_S4: Expression profile of the top genes within the integrated dataset ……………………………………………………………………………………20-21

Supplemental_Fig_S5: Individual expression UMAP plots of the top 3 genes used to annotate cellular populations ……………………………………………..……...22-23

Supplemental_Fig_S6: Differentially regulated enrichment terms in tau P251L KI brains compared to controls ……………………………………………………**24-25**

Supplemental_Fig_S7: Differential gene expression in the central body, optic lobe, and glia in tau P251L knock-in brains compared to controls …………..**26-27**

Supplemental_Fig_S8: Protein interaction networks enriched in the central body of tau P251L KI brains………………………………………………………………..**28-29**

Supplemental_Fig_S9: Protein interaction networks enriched in the optic lobe of tau P251L KI brains ……………………………………………………………………..**30-31**

Supplemental_Fig_S10: Protein interaction networks enriched in glial cells of tau P251L KI brains …………………………………………………………………………..**32-33**

Supplemental_Fig_S11: Compared to control, cell-cell communication analysis of tau P251L KI brains reveals a distinctive role of glial and Kenyon cells ………………………………………………………………………………………..**34-35**

Supplemental_Fig_S12: Differential gene expression and enrichment analysis in the Kenyon cells (KC) of tau P251L KI brains compared to controls …………..**36-37**

Supplemental_Fig_S13: Density plots compare the expression of regulons enriched in tau P251L knock-in Kenyon cells compared to controls …………**38-39**

Supplemental References ……………………………………………………………**40-42**

Supplemental Methods

Sectioning, immunostaining and imaging

Adult flies were fixed in formalin at 1, 10 and 30 days of age and embedded in paraffin. Serial frontal sections including the entire brain were prepared. Sections were stained with hematoxylin and eosin to assess brain vacuolization, which was quantified by counting vacuoles larger than 5 microns throughout the entire brain. For immunostaining, antigen retrieval was performed by microwaving the sections in 10 mM sodium citrate buffer. Immunohistochemistry was performed with the avidin–biotin–peroxidase complex detection method (Vector Laboratories). For immunofluorescence, secondary antibodies coupled to Alexa 488 or Alexa 555 (Invitrogen, 1:200) were used and sections were mounted in DAPI containing mounting media. The number of PCNA-positive cells throughout the entire brain was counted following immunostaining. For quantification of pH2Av, a region of interest comprised of approximately 100 Kenyon neurons was identified in well-oriented sections of the mushroom body and the number of neurons containing one or more than one immuno-positive foci was determined. Images were taken on Zeiss LSM800 confocal microscope (Carl Zeiss, AG), and quantification was performed using Image-J software. For all histological analyses, at least 6 brains were analyzed per genotype and time point. The sample size (n), mean and SEM are given in the figure legends. All statistical analyses were performed using GraphPad Prism 5.0. For comparisons across more than 2 groups, one-way ANOVA with Tukey post-hoc analysis was used. For comparison of 2 groups Student's *t*-tests were performed.

Comet assay

Two brains per genotype were dissected from adult flies in ice cold PBS. The brains were homogenized with a plastic pestle and subjected to comet using commercially available reagents (CometAssay, Trevigen). Fifty nuclei were quantified per trail using Casplab software. The experiment was repeated 3 times.

Measurement of oxygen consumption and extracellular acidification rates

The OCR and extracellular acidification rate were measured as previously described (Sarkar et al. 2020). Briefly, brains from 10-day-old flies were dissected and plated at one brain per well on XFe96 plates (Seahorse Bioscience) and metabolic parameters were assayed. 6 brains per genotype were analyzed. OCR values were normalized to DNA content using a CyQUANT assay (Thermo Fisher Scientific) following the manufacturer's protocol.

scRNA sequencing: sample preparation

To dissociate fly brains for the scRNA sequencing we modified previously published fly brain dissociation protocols (Li et al. 2017; Davie et al. 2018). Briefly, 20 male and 20 female brains from 10-day-old flies were dissected on ice cold Schneider's medium with FBS (Gibco, filtered 10% FBS). After a brief centrifugation, the supernatant was removed and the brains washed with ice cold PBS to remove Schneider's medium. The brains were then incubated at 25 °C with 300 μl of 0.05% trypsin-EDTA (Fisher Scientific) for 30 minutes, with continuous pipetting every 5 minutes. Additionally, the solution containing brain chunks was passed through 25-gauge needle (25G 5/8), without introducing air

bubbles, roughly 50 times. After the brains were fully dissociated, the resultant solution was poured through a 10 μm pluri-select cell strainer (Fisher Scientific) and 400 μl of ice-cold Schneider's medium containing FBS was added to inactivate trypsin. The sample was centrifuged for 15 minutes at 600 x g and the supernatant was removed without disturbing the pellet. The pellet was suspended in sterile PBS containing 0.04% BSA. The cells were quantified, and the viability was determined using AO-PI reagents (Logos Biosystems).

Single-cell encapsulation, sequencing, and downstream processing

We proceeded only with samples having more than 90% viability for the single-cell encapsulation. The samples were encapsulated, 6 libraries were prepared, 3 control and 3 tau P251L knock-in, at the single-cell core facility at Harvard Medical School, following the manufacturer's protocol (10x Genomics). The libraries were sequenced on NovaSeq 6000 V1.5 S2 located in the Harvard Medical School Biopolymers Facility.

10x raw data processing

The sequenced libraries were processed using Cellranger (version 6.0, 10x Genomics). The *Drosophila* reference genome BDGP6.32 was used and built following 10x Genomics users guide instruction. The output of Cellranger were used as an input of SoupX (version 1.5.2) and Scrublet (version 0.2.2) to remove potential ambient RNA and doublets, respectively. The resulting count matrices, indicating transcripts (UMI) and cells (barcodes) detected by sequencing, were used for quality control and downstream analysis.

Seurat data processing

The ambient RNA and doublet removed count matrices were used as input of Seurat in R (version 4.1.0) (Team 2016; Butler et al. 2018). For each library, cells with less than 10% of mitochondrial genes were kept for upstream analysis. In addition, cells with feature counts and UMI counts within 3 standard deviations of the mean library feature counts and UMI counts were kept for downstream processing. In addition, only features detected in at least 3 cells were kept. Data normalization was then performed across libraries to remove variation of sequencing depth, by employing a global-scaling normalization method "LogNormalize" (i.e., the feature expression measurements for each cell were divided by the total expression, multiplied by a scale factor of 10,000, and then log-transformed). The top 2,000 most variable features were selected and used for downstream analysis including dimension reduction. To annotate cell types and perform differential expression analysis between conditions in an experiment, libraries sequenced in one experiment were integrated by identifying common anchors between conditions. The dimensions of the expression matrices were then reduced by principal component analysis (PCA). The ElbowPlot function was used to determine the optimal number of dimensions used to identify cell clusters. Cell clusters were identified with the default method in Seurat. In brief, a K nearest neighbors (KNN) graph was first constructed based on the Euclidean distance of cells in PCA space, and the edge weights between any two cells were refined based on the shared overlap in their local neighborhoods (Jaccard similarity). Louvain algorithm was applied to iteratively group cells together to form optimized communities with the resolution parameter of 0.5. Cells within the graph-based clusters determined above were further visualized and explored by Uniform Manifold

Approximation and Projection (UMAP) non-linear dimensional reduction (Becht et al. 2019). The cell clusters were then annotated based on manual inspection of top feature genes and known marker genes expression in each cluster. Finally, the differentially expressed genes across conditions were examined by comparing expression profile of each cluster across experimental conditions using MAST 1.23.1 (Finak et al. 2015). The significant differentially expressed genes (DEGs) were defined as FDR-adjusted p-value < 0.05 and absolute $\log_2$fold-change > 0.25. The top DEGs were also shown in the volcano plots and the heatmap.

Cell cluster annotation, gene enrichment, and Ontology analyses

To annotate cluster identity, we used data collated by the *Drosophila* RNAi Screening Center single-cell RNA sequencing DataBase (DRscDB) from previous *Drosophila* brain single cell sequencing analyses (Davie et al. 2018; Hu et al. 2021; Li et al. 2022). Gene enrichment and Ontology analyses were performed with FlyEnrichr(Chen et al. 2013; Kuleshov et al. 2016) using 2 differentially enriched gene lists: 1) differentially up-regulated genes, FDR-adjusted p-value < 0.05 and $\log_2$fold-change > 0.25, throughout all brain cell clusters, and 2) differentially down-regulated genes, FDR-adjusted p-value < 0.05 and $\log_2$fold-change > -0.25 throughout all cell clusters in the tau P251L *Drosophila* brain. GeneRIF terms were used for Gene Ontology analysis. We showed the top 5 enriched terms for all the gene enrichment analysis and removed the redundant terms in the same gene sets. Enrichment analysis data is presented as a combined score, which is the combination of the p-value (computed using Fisher exact test) and z-score (computed to assess the deviation from the expected rank) calculated by multiplying the two scores, giving the combined score (c) = log(p)*z (Chen et al. 2013). Gene enrichment

terms, genes, genes contributing to the identified enrichment terms, p-values, z-scores, and combined scores are given in each supplementary files and displayed in the corresponding figures.

Protein-protein interaction network and cell-cell communication analysis

For the protein-protein network analysis, all the annotated clusters in the single-cell RNA sequencing dataset were classified based on their location within the *Drosophila* brain: central body, optic lobe, and glial cells. Differentially expressed genes with a $\log_2$ fold change > 0.25 and < -0.25 with FDR < 0.05, were used as input for the OmicsIntegrator protein-protein interaction package (Tuncbag et al. 2016). Fold change was used as the prize. Network hyperparameters were sorted hierarchically by the average node specificity, node robustness, and KS statistic of the degree of predicted nodes to that of prize nodes to choose a parameter set. In short, robustness was determined as the percentage of times a node appeared in the network after 100 random permutations of the network edges. Node specificity was the percentage of networks in which the node was observed after randomly shuffling the prize values and re-running the algorithm 100 times. A network parameter set corresponding to smaller specificity values, higher robustness values, and smaller KS statistics was selected. The genes identified in each network were used to annotate the biological processes using FlyEnrichr (Kuleshov et al. 2016). The cell-cell interaction analysis was performed as previously published (Liu et al. 2022).

Trajectory analysis

Trajectory analysis was performed using Slingshot (Street et al. 2018) on astrocyte subclusters. Trajectories were calculated on defined subclusters with the cell type

showing the highest entropy used as a starting cluster (Guo et al. 2017). Differential gene expression along each pseudotime lineage was calculated using a generalized additive model (Chambers and Hastie 2017). The first 1000 genes from all lineages were calculated and top 100 genes were shown on the heatmaps.

Gene regulatory network analyses

The previously published pySCENIC (v 0.10.0) (Single-Cell rEgulatory Network Inference and Clustering) pipeline with the *Drosophila* genome 9 (Dm9) reference genome was used to assess gene regulatory networks (Van de Sande et al. 2020). The level of regulon activity in each cell was scored using AUCell, which was then converted to a binary scale to reflect the presence or absence of the regulon.

Supplemental Tables

Supplemental Table S1: Top 10 markers of the identified clusters, number of cells, and types of cells in the final integrated dataset. Refer to Supplemental_Table_S1.xlsx

Supplemental Table S2: Normalized read counts of the identified genes in all the clusters. Refer to Supplemental_Table_S2.xlsx

Supplemental Table S3: Differentially regulated genes (DEGs) and Gene Ontology terms in the P251L KI brains. Refer to Supplemental_Table_S3.xlsx

Supplemental Table S4: Up- and down-regulated genes and Gene Ontology terms in the major cluster types in tau P251L KI brains. Refer to Supplemental_Table_S4.xlsx

Supplemental Table S5: Up- and down-regulated DEGs and Gene Ontology in glial cell clusters and pseudotime DEGs in astrocyte clusters. Refer to Supplemental_Table_S5.xlsx

Supplemental Table S6: Up- and down-regulated DEGs and Gene Ontology in Kenyon cell clusters of tau P251L KI brains compared to controls. Refer to Supplemental_Table_S6.xlsx

Supplemental Table S7: Details of regulons identified, via gene regulatory network analysis, in control and tau P251L KI Kenyon cells. Refer to Supplemental_Table_S7.xlsx

Supplemental Figures

```
H. Sapiens         MAEPRQEFEVMEDHAGTYGLGDRKDQGG-YTMHQDQEGD---TDAGLKESPLQTPTEDGSEE 58
M. Musculus        MADPRQEFDTMEDHAGDY------------TLLQDQEGD---MDHGLKESPPQPPADDGAEE 47
B. Tarus           MAEPRQEFDVMEDHAQ----------GD-YT-LQDQEGD---MDPGLKESPLQTPADDGSEE 47
C. Elegans(PTL1)   MSTPQSEPGSEPGSP-----------GENENSMETQYSQGSFPEVAVEPEPEPEP------E 45
D. Melanogaster    MAD-VLEKSSLLDAVPP--------LGDPHPPLPHQQLQ---QEAAAA-------------- 36

H. Sapiens         PGSETSDAKSTPTAEDVTAPLVD---EGAPGKQAAAQP-HTEIPEGTTAEEAGIG-DTPSLE 115
M. Musculus        PGSETSDAKSTPTAEDVTAPLVD---ERAPDKQAAAQP-HTEIPEGITAEEAGIG-DTPNQE 104
B. Tarus           PGSETSDAKSTPTAEDATAPLVD---EGAPGEQAAAQA-PAEIPEGTAAEEAGIG-DTSNLE 104
C. Elegans(PTL1)   PEPE-------PEPE----P------EPEP--------EPEPKPK---PEVEPVPQPEPEPE 79
D. Melanogaster    ----------------------------------------------AAANAA-PPAPPQQ 49

H. Sapiens         DEAAGHVTQARMVSKSKDGTGSDDKKAKGADGKT--KIATPRGAAPPG--QKGQANATRIPA 173
M. Musculus        DQAAGHVTQARVASKD--RTGNDEKKAKGADGKTGAKIATPRGAASPA--QKGTSNATRIPA 162
B. Tarus           DQAAGHVTQARMVSKGKDGTGPDDKKTKGADGKPGTKIATPRGAAPPG--QKGQANATRIPA 164
C. Elegans(PTL1)   YQPEPEPEPEVEPEPEPEPVPEPEPEP-EPEPEPVVEKEEEVVVESPPREQEPEKSGKSKPS 140
D. Melanogaster    QQPPPHQLQQQQPQQQQLQQKPANARANQD-QKEG---DNDSGVDEST--QEKDRNGPNSPS 105

H. Sapiens         KTPPAPKT----------PP--SS----GEPPKSGDRSGYSSP------------------- 200
M. Musculus        KTTPSPKT----------PP--GS----GEPPKSGERSGYSSP------------------- 189
B. Tarus           KTTPTPKTSPATMQVQKKPPPAGAKSERGESGKSGDRSGYSSP------------------- 207
C. Elegans(PTL1)   SPIPDA------------PTMEDIAPRELESLNFSETSGTSDQQADRIMQNNENERVEEKKQ 190
D. Melanogaster    SPV-KT------------PT-ST-----SSKP---DKSGTSRP------------------- 126

H. Sapiens         --GSPGTPGSRSRTPSLPTPPT---REPKK-VAVVRTP----PK-SPSSAKSRLQTA---PV 248
M. Musculus        --GSPGTPGSRSRTPSLPTPPT---REPKK-VAVVRTP----PK-SPSASKSRLQTA---PV 237
B. Tarus           --GSPGTPGSRSRTPSLPTPPT---REPKK-VAVVRTP----PK-SPSAAKSRLQAA---PG 255
C. Elegans(PTL1)   MSPTPSQPQHKTPQRSGIRPPTAILRQPKPIPASLPRPATAT-PSSQRAISTPRQTASTAPS 251
D. Melanogaster    --PS-ATPSNKS------APKS---RSASKNRLLLKTPE---PEPVKKVPMNKVQVG---HA 170

                                                         MTBD repeat
H. Sapiens         PMPD--------------------------------LKNVKSKIGSTENLKHQPGGG------ 273
M. Musculus        PMPD--------------------------------LKNVRSKIGSTENLKHQPGGG------ 262
B. Tarus           PMPD--------------------------------LKNVKSKIGSTENLKHQPGGG------ 280
C. Elegans(PTL1)   PRPISKMSRERSDVQKSTSTRSIDNVGRMTPKVNAKFVNVKSKVGSVTN--HKAGGGNVEIF 311
D. Melanogaster    PSPN--------------------------------LKAVRSKIGSLDNATYKPGGGHVKIE 200

                                                         MTBD repeat
H. Sapiens         --------------------------KVQIINKKLDLSNVQSKCGSKDNIKHVPGGGSVQIV 309
M. Musculus        --------------------------KVQIINKKLDLSNVQSKCGSKDNIKHVPGGGSVQIV 298
B. Tarus           --------------------------KVQIINKKLDLSNVQSKCGSKDNIKHVPGGGSVQIV 316
C. Elegans(PTL1)   SEKRLY-NAQSKVGSLKNATHVAGGGNVQIENRKLDFSAASPKVGSKTN--YQPAKSDVKIV 370
D. Melanogaster    SKKIDIKAAPRIEA--KNDKYMPKGGEKKIVTTKL-QWNAKSKIGSLENAAHKPGGGDKKIE 259

                      MTBD repeat                            MTBD repeat
H. Sapiens         YKPVDLSKVT-SKCGSLGNIHHKPGGGQVEVKSEKLDFKDRVQSKIGSLDNITHVPGGGNKK 370
M. Musculus        YKPVDLSKVT-SKCGSLGNIHHKPGGGQVEVKSEKLDFKDRVQSKIGSLDNITHVPGGGNKK 359
B. Tarus           YKPVDLSKVT-SKCGSLGNIHHKPGGGQVEVKSEKLDFKDRVQSKIGSLDNITHVPGGGNKK 316
C. Elegans(PTL1)   SEKL-TWQAK-SKVGSMDNAAHKPAGGNVQILSQKL-NW-KAESKVGSKDNMNHRPGGGNVQ 428
D. Melanogaster    TLKMDFKDKAKPKVGSTANVKHQPGGGDIKIQTQKLEIKA--QSKVGSLDNVKHKPGGGEKK 319

H. Sapiens         IETHKLTFRENAKAKTDHGAEIVYKSPVVSGDTSPRHLSNVSSTGSIDMVDSPQLATLADEV 432
M. Musculus        IETHKLTFRENAKAKTDHGAEIVYKSPVVSGDTSPRHLSNVSSTGSIDMVDSPQLATLADEV 421
B. Tarus           IETHKLTFRENAKAKTDHGAEIVYKSPVVSGDTSPRHLSNVSSTGSIDMVDSPQLATLADEV 439
C. Elegans(PTL1)   IFDEKIRYVSTDSSRNHSTLDISSL------------------------------------- 453
D. Melanogaster    IFDDKDYLKNVEHSVALTTPPTQSPLPSMTASGADENLNQQS-------------------- 361

H. Sapiens         SASLAKQGL       441
M. Musculus        SASLAKQGL       430
B. Tarus           SASLAKQGL       448
C. Elegans(PTL1)   ---------       453
D. Melanogaster    ---------       361
```

Supplemental Figure S1: Multiple sequence alignment of tau across species. Alignment of human tau (4 repeat) and C. elegans PTL1 (tau homolog) to tau from different species. Conserved residues are highlighted in bold, and human proline 301 and orthologous prolines in other species are shown in bold red. The alignment highlights conservation of microtubule binding domains (MTBD).

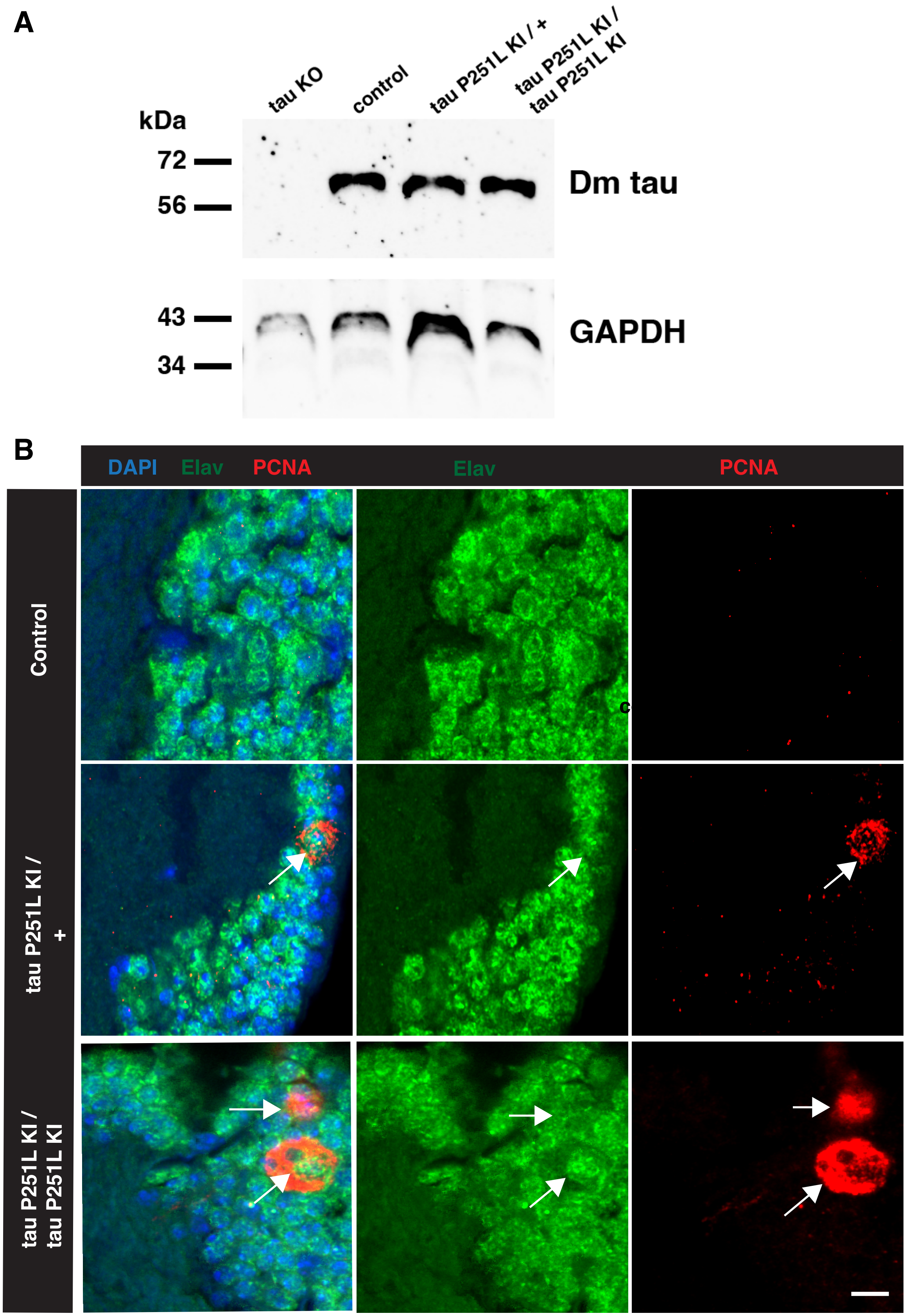
A
tau KO
control
tau P251L KI / +
tau P251L KI / tau P251L KI
kDa
72
56
Dm tau
43
34
GAPDH
B
DAPI Elav PCNA
Elav
PCNA
Control
tau P251L KI / +
tau P251L KI / tau P251L KI

Supplemental Figure S2: *Drosophila* tau levels and PCNA staining in tau P251L KI brains compared to controls. Immunoblotting analysis using an antibody to *Drosophila* tau shows equivalent levels of wild type and P251L tau (*A*). The blot is reprobed with an antibody to GAPDH to illustrate equivalent protein loading. Representative images of proliferating cell nuclear antigen staining in control and tau P251L knock-in Kenyon cells (arrows, identified with the neuronal marker elav) (*B*). Control is *elav-GAL4/+*. Flies are 10 days old in (*A)* and 30 days old in (*B*).

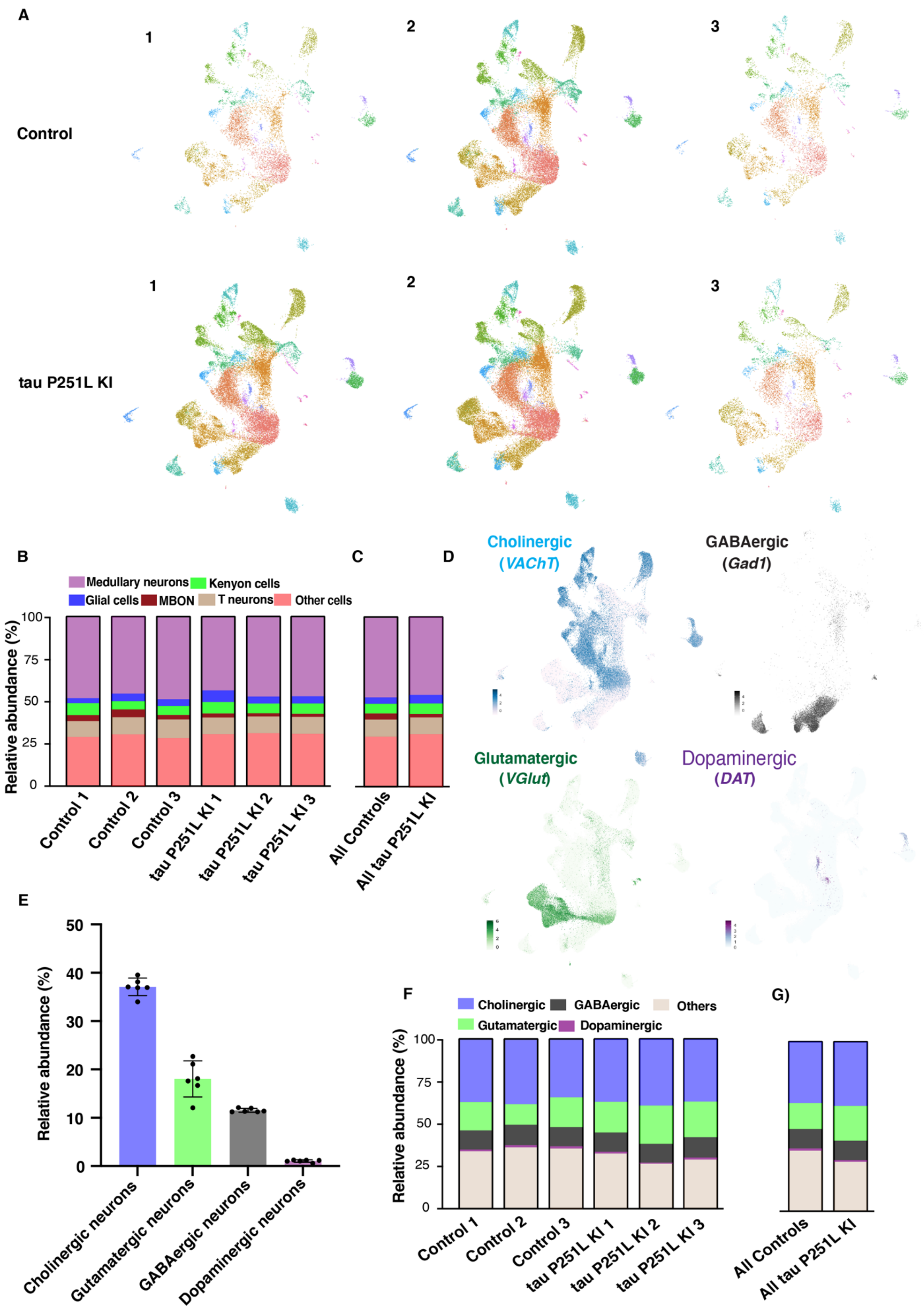

A
Control
tau P251L KI
1
2
3
B
C
D
Medullary neurons
Kenyon cells
Glial cells
MBON
T neurons
Other cells
Relative abundance (%)
Control 1
Control 2
Control 3
tau P251L KI 1
tau P251L KI 2
tau P251L KI 3
All Controls
All tau P251L KI
Cholinergic
(*VAChT*)
GABAergic
(*Gad1*)
Glutamatergic
(*VGlut*)
Dopaminergic
(*DAT*)
E
Cholinergic neurons
Gutamatergic neurons
GABAergic neurons
Dopaminergic neurons
F
G)
Cholinergic
GABAergic
Others
Gutamatergic
Dopaminergic

Supplemental Figure S3: Relative abundance of the cells and neuronal identities within the final integrated dataset. Individual 10x runs were integrated and contained cells from all clusters: 3 of control and 3 of tau P251L KI and relative abundance (%) of the medullary neurons (14 clusters), Kenyon cells (3 clusters), glial cell (2 clusters), MBON cluster, and T-neurons (3 clusters) in the individual scRNA-seq runs (*A,B*). Relative abundance (%) of the 3 integrated control and tau P251L KI datasets (*C*). Relative expression (>2 fold) of the key neuronal identity markers genes, such as *VAChT* for cholinergic neurons, *Gad1* for GABAergic neurons, *VGlut* for glutamatergic neurons, and *DAT* for dopaminergic neurons, within the integrated dataset (*D*). Relative abundance (%) of the neuronal identities within the integrated dataset (*E*). Relative abundance (%) of the neuronal identities in the individual scRNA-seq runs (*F*). Relative abundance (%) of neuronal identities in the 3 integrated control and tau P251L KI datasets (*G*).

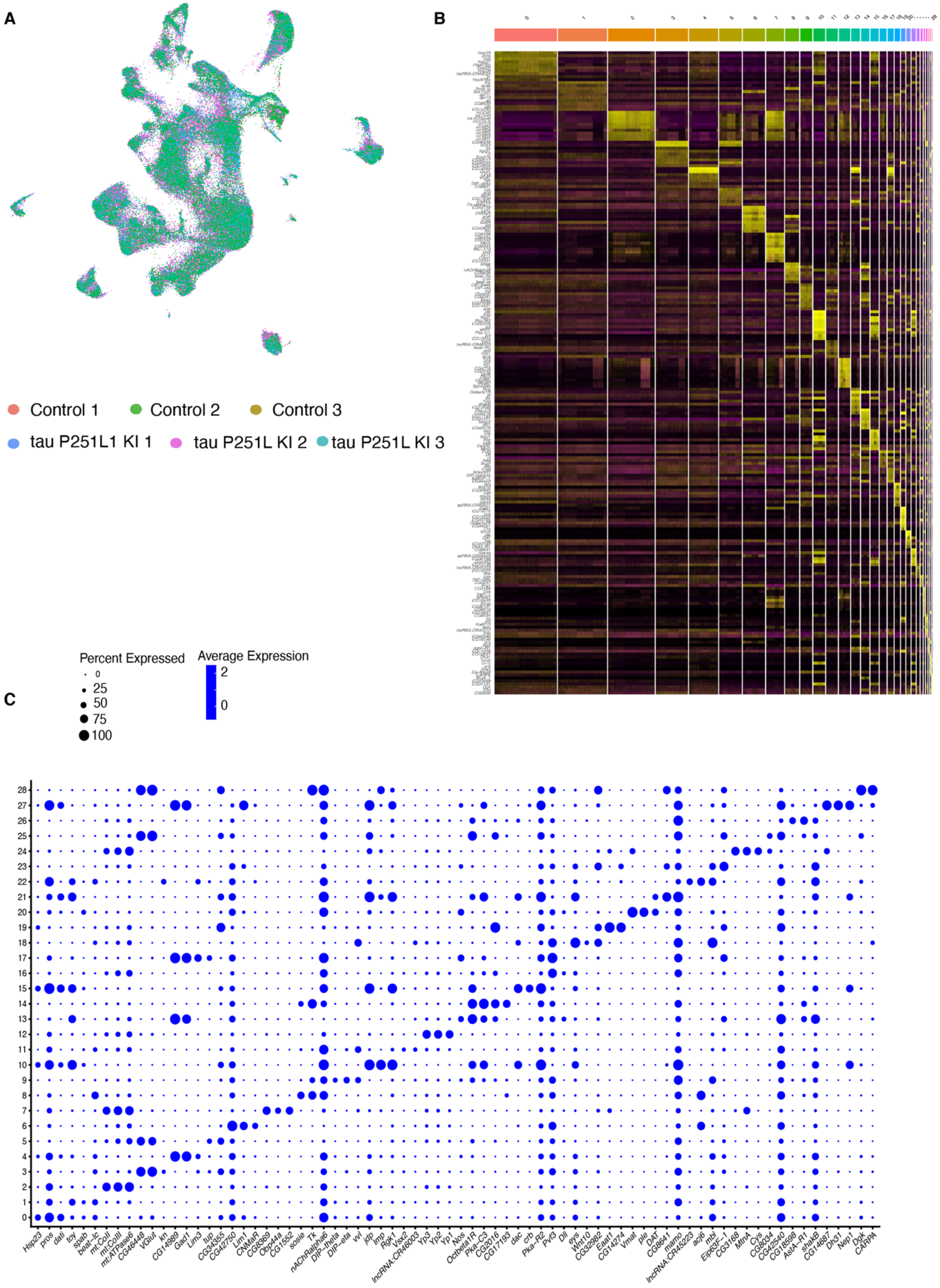
A
Control 1
Control 2
Control 3
tau P251L1 KI 1
tau P251L KI 2
tau P251L KI 3
B
C
Percent Expressed
0
25
50
75
100
Average Expression
2
0
Hsp23 pros dati toy spab beat-Ic mt:CoII mt:CoIII mt:ATPase6 CG46448 VGlut kn CG14989 Gad1 Lim3 tup CG34355 CG42750 Lim1 CNMaR CG8369 Obp44a CG1552 sosie Tk nAChRalpha6 DIP-theta DIP-eta vvl jdp Imp Rgk1 Vsx2 lncRNA:CR46003 Yp3 Yp2 Yp1 Nos Octbeta1R Pka-C3 CG2016 CG17193 dac crb Pka-R2 Pvf3 Dll eys Wnt10 CG32982 Eaat1 CG14274 Vmat ple DAT CG8641 mamo lncRNA:CR45223 acj6 mbl Eip63F-1 CG3168 MtnA Cys CG8034 CG42540 CG18598 AstA-R1 shakB CG14687 Dh31 Nep1 Dgk CARPA

Supplemental Figure S4: Expression profile of the top genes within the integrated dataset. The proportion of each sample, 3 of control and 3 of tau P251L KI, within the final integrated dataset obtained after scRNA-seq bioinformatics pipeline *(A)*. Heatmap of the top 10 highly expressed genes emerged after dimensionality reduction in all the clusters within the integrated dataset *(B)*. A dot plot showing the percentage expression of the top 3 genes within each single cell cluster identified in the integrated dataset. These 3 top markers and other top 7 markers (top 10 markers) were used to annotate the single cell cluster identities *(C)*.

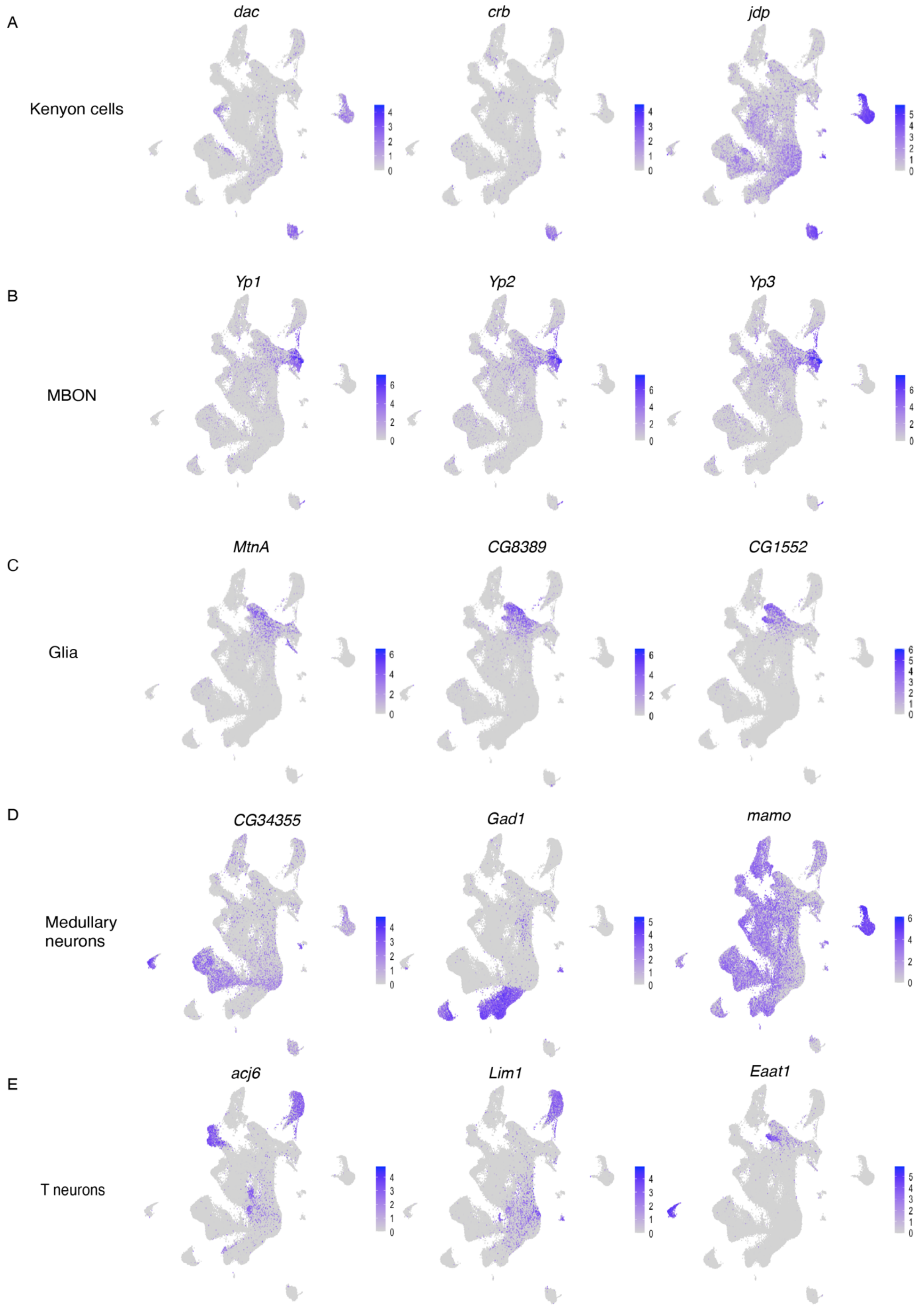
A
Kenyon cells
dac
crb
jdp
B
MBON
Yp1
Yp2
Yp3
C
Glia
MtnA
CG8389
CG1552
D
Medullary neurons
CG34355
Gad1
mamo
E
T neurons
acj6
Lim1
Eaat1

Supplemental Figure S5: Individual expression UMAP plots of the top 3 genes used to annotate cellular populations. Relative expression of the top marker genes, such as *dac*, *crb*, and *jdp*, within the integrated dataset used to annotate Kenyon cells *(A)*. Relative expression of top marker genes, such as *Yp1*, *Yp2*, and *Yp3*, within the integrated dataset used to annotate mushroom body output neurons (MBON) *(B)*. Relative expression of top marker genes, such as *MtnA*, *CG8369*, and CG1522, within the integrated dataset annotating the glial cells *(C)*. Relative expression of top marker genes, such as *CG34355*, *Gad1*, and *mamo*, within the integrated dataset, used to annotate the medullary neurons *(D)*. Relative expression of top marker genes, such as *acj6*, *Lim1*, and *sosie*, within the integrated dataset, used to annotate the T neurons *(E)*.

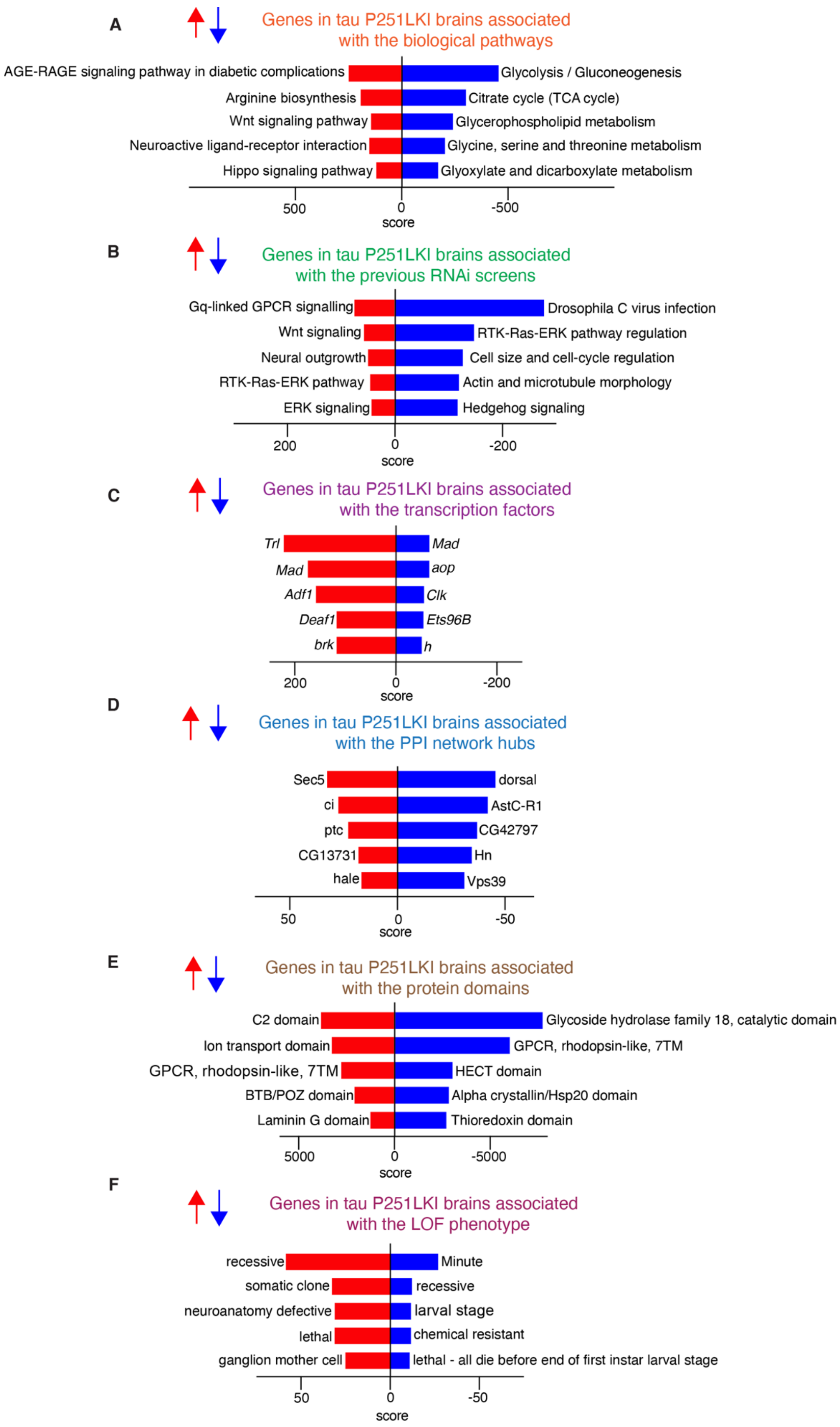
A
Genes in tau P251LKI brains associated with the biological pathways
AGE-RAGE signaling pathway in diabetic complications
Arginine biosynthesis
Wnt signaling pathway
Neuroactive ligand-receptor interaction
Hippo signaling pathway
Glycolysis / Gluconeogenesis
Citrate cycle (TCA cycle)
Glycerophospholipid metabolism
Glycine, serine and threonine metabolism
Glyoxylate and dicarboxylate metabolism
500
0
-500
score
B
Genes in tau P251LKI brains associated with the previous RNAi screens
Gq-linked GPCR signalling
Wnt signaling
Neural outgrowth
RTK-Ras-ERK pathway
ERK signaling
Drosophila C virus infection
RTK-Ras-ERK pathway regulation
Cell size and cell-cycle regulation
Actin and microtubule morphology
Hedgehog signaling
200
0
-200
score
C
Genes in tau P251LKI brains associated with the transcription factors
Trl
Mad
Adf1
Deaf1
brk
Mad
aop
Clk
Ets96B
h
200
0
-200
score
D
Genes in tau P251LKI brains associated with the PPI network hubs
Sec5
ci
ptc
CG13731
hale
dorsal
AstC-R1
CG42797
Hn
Vps39
50
0
-50
score
E
Genes in tau P251LKI brains associated with the protein domains
C2 domain
Ion transport domain
GPCR, rhodopsin-like, 7TM
BTB/POZ domain
Laminin G domain
Glycoside hydrolase family 18, catalytic domain
GPCR, rhodopsin-like, 7TM
HECT domain
Alpha crystallin/Hsp20 domain
Thioredoxin domain
5000
0
-5000
score
F
Genes in tau P251LKI brains associated with the LOF phenotype
recessive
somatic clone
neuroanatomy defective
lethal
ganglion mother cell
Minute
recessive
larval stage
chemical resistant
lethal - all die before end of first instar larval stage
50
0
-50
score

Supplemental Figure S6: Differentially regulated enrichment terms in tau P251L KI brains compared to controls. KEGG pathways analysis of all up-regulated genes in tau P251L KI brains identified the AGE-RAGE signaling pathway in diabetic complications and the glycolysis pathway for the down-regulated genes in tau P251L KI brains *(A)*. RNAi screen from genome RNAi identified Gq-linked GPCR signaling RNAi screen for the up-regulated genes in tau P251L KI brains and *Drosophila* C virus infection screen for the down-regulated in tau P251L KI brains *(B)*. The transcription factor analysis identified most of the up-regulated genes in tau P251L KI brains as associated with the *Trl* transcription factor and down-regulated genes in tau P251L KI brains as associated with the *Mad* transcription factor *(C)*. Protein-protein interactions network hub analysis identified up-regulated genes in tau P251L KI brains associated with the Sec5 and down-regulated genes in tau P251L KI brains to be associated with dorsal PPI network hub *(D)*. InterPro domain analysis identified the C2 domain as associated with the up-regulated genes in tau P251L KI brains and the Glycoside hydrolase family 18 as associated with the down-regulated genes in tau P251L KI brains *(E)*. Loss of function (LOF) phenotype analysis identified the term recessive associated with the up-regulated genes in tau P251L KI brains and minute associated with the down-regulated genes in tau P251L KI brains *(F)***.** score = log(p)*z.

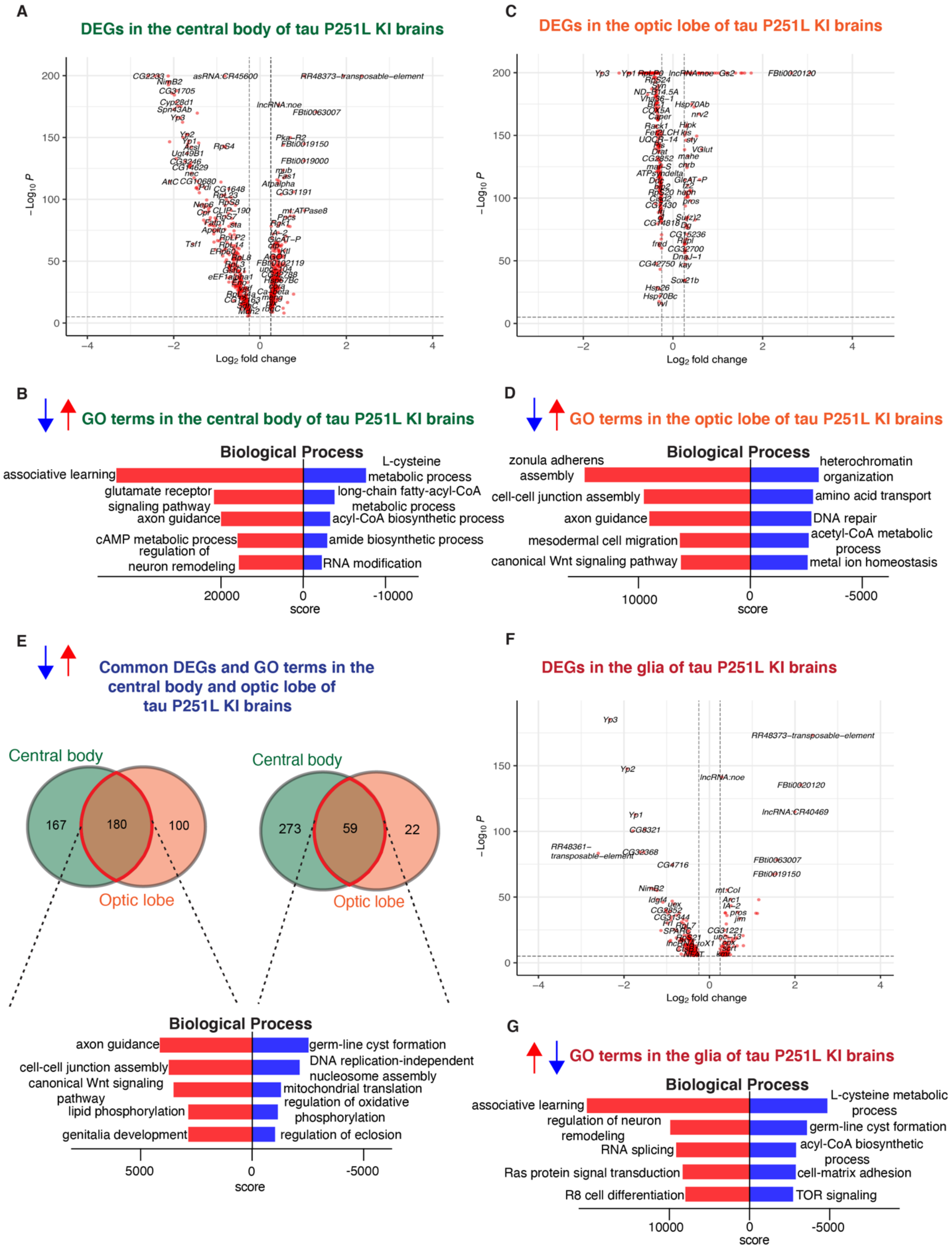
A
DEGs in the central body of tau P251L KI brains
−Log10 P
Log2 fold change
B
GO terms in the central body of tau P251L KI brains
Biological Process
associative learning
glutamate receptor signaling pathway
axon guidance
cAMP metabolic process
regulation of neuron remodeling
L-cysteine metabolic process
long-chain fatty-acyl-CoA metabolic process
acyl-CoA biosynthetic process
amide biosynthetic process
RNA modification
20000
0
-10000
score
C
DEGs in the optic lobe of tau P251L KI brains
−Log10 P
Log2 fold change
D
GO terms in the optic lobe of tau P251L KI brains
Biological Process
zonula adherens assembly
cell-cell junction assembly
axon guidance
mesodermal cell migration
canonical Wnt signaling pathway
heterochromatin organization
amino acid transport
DNA repair
acetyl-CoA metabolic process
metal ion homeostasis
10000
0
-5000
score
E
Common DEGs and GO terms in the central body and optic lobe of tau P251L KI brains
Central body
Optic lobe
167
180
100
273
59
22
Biological Process
axon guidance
cell-cell junction assembly
canonical Wnt signaling pathway
lipid phosphorylation
genitalia development
germ-line cyst formation
DNA replication-independent nucleosome assembly
mitochondrial translation
regulation of oxidative phosphorylation
regulation of eclosion
5000
0
-5000
score
F
DEGs in the glia of tau P251L KI brains
−Log10 P
Log2 fold change
G
GO terms in the glia of tau P251L KI brains
Biological Process
associative learning
regulation of neuron remodeling
RNA splicing
Ras protein signal transduction
R8 cell differentiation
L-cysteine metabolic process
germ-line cyst formation
acyl-CoA biosynthetic process
cell-matrix adhesion
TOR signaling
10000
0
-5000
score

Supplemental Figure S7: Differential gene expression in the central body, optic lobe, and glia in tau P251L knock-in brains compared to controls. Differentially regulated genes, both up-and down-regulated, in the central body of tau P251L knock-in brains *(A)*. GO analysis shows biological processes associated with up-regulated and down-regulated genes in the central body of tau P251L knock-in brains *(B)*. Differentially regulated genes, both up and down-regulated, in the optic lobe of tau P251L knock-in brains *(C)*. GO analysis shows biological processes associated with up-regulated and down-regulated genes in the optic lobe of tau P251L knock-in brains *(D)*. Biological processes, up and down-regulated, in the common neuronal genes in the central body and optic lobe *(E)*. Differentially regulated genes, both up and down-regulated, in glia of tau P251L knock-in brains *(F)*. GO analysis shows biological processes associated with up-regulated and down-regulated genes in the glia of tau P251L knock-in brains *(G)*. All dots on the volcano plots are significant at FDR < 0.05 and $\log_2$fold-change > 0.25 for up-regulated and < -0.25 for down-regulated genes. The score represents the combined score c = log(p)*z (Chen et al. 2013).

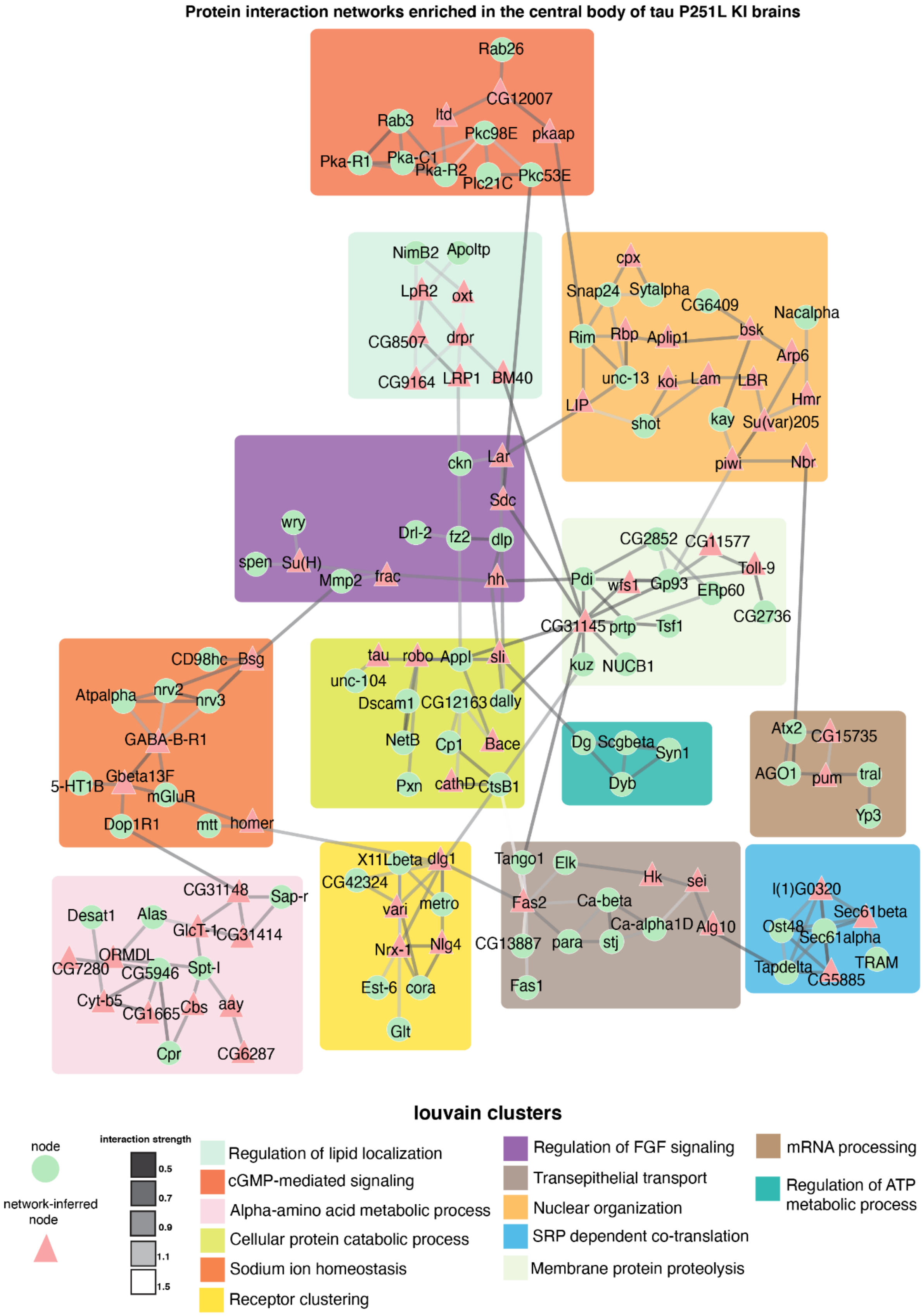
Protein interaction networks enriched in the central body of tau P251L KI brains
Rab26
CG12007
Rab3
ltd
Pkc98E
pkaap
Pka-R1
Pka-C1
Pka-R2
Plc21C
Pkc53E
NimB2
Apoltp
LpR2
oxt
CG8507
drpr
CG9164
LRP1
BM40
cpx
Snap24
Sytalpha
CG6409
Nacalpha
Rim
Rbp
Aplip1
bsk
Arp6
unc-13
koi
Lam
LBR
LIP
Hmr
shot
kay
Su(var)205
piwi
Nbr
ckn
Lar
Sdc
wry
Drl-2
fz2
dlp
spen
Su(H)
Mmp2
frac
hh
CG2852
CG11577
Toll-9
Pdi
wfs1
Gp93
ERp60
CG31145
prtp
Tsf1
CG2736
kuz
NUCB1
CD98hc
Bsg
Atpalpha
nrv2
nrv3
GABA-B-R1
5-HT1B
Gbeta13F
mGluR
Dop1R1
mtt
homer
tau
robo
Appl
sli
unc-104
Dscam1
CG12163
dally
NetB
Cp1
Bace
Pxn
cathD
CtsB1
Dg
Scgbeta
Syn1
Dyb
Atx2
CG15735
AGO1
pum
tral
Yp3
CG31148
Sap-r
Desat1
Alas
GlcT-1
CG31414
ORMDL
CG7280
CG5946
Spt-I
Cyt-b5
CG1665
Cbs
aay
Cpr
CG6287
X11Lbeta
dlg1
CG42324
vari
metro
Nrx-1
Nlg4
Est-6
cora
Glt
Tango1
Elk
Hk
sei
Fas2
Ca-beta
Ca-alpha1D
Alg10
CG13887
para
stj
Fas1
l(1)G0320
Sec61beta
Ost48
Sec61alpha
Tapdelta
TRAM
CG5885
louvain clusters
node
network-inferred node
interaction strength
0.5
0.7
0.9
1.1
1.5
Regulation of lipid localization
cGMP-mediated signaling
Alpha-amino acid metabolic process
Cellular protein catabolic process
Sodium ion homeostasis
Receptor clustering
Regulation of FGF signaling
Transepithelial transport
Nuclear organization
SRP dependent co-translation
Membrane protein proteolysis
mRNA processing
Regulation of ATP metabolic process

Supplemental Figure S8: Protein interaction networks enriched in the central body of tau P251L KI brains. OmicsIntegrator identified various PPI interaction maps in the central body of tau P251L KI brains, including regulation of lipid localization, cGMP-mediated signaling, alpha-amino acid transport, cellular protein catabolic process, sodium ion homeostasis, receptor clustering, regulation of FGF signaling, transepithelial transport, nuclear organization, SRP dependent co-translation, membrane protein proteolysis, mRNA processing, and electron transport chain.

Protein interaction networks enriched in the optic lobe of tau P251L KI brains

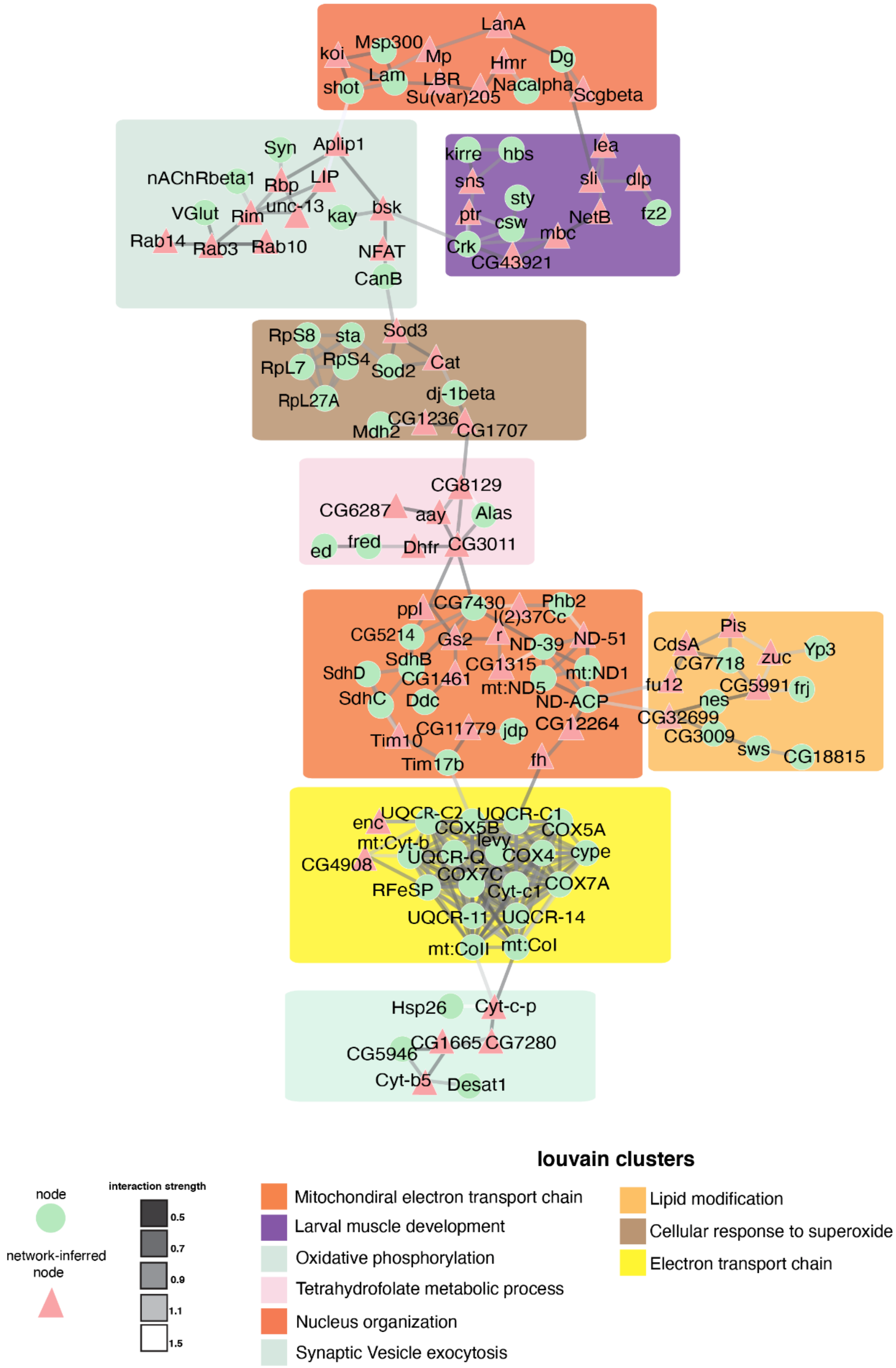

Supplemental Figure S9: Protein interaction networks enriched in the optic lobe of tau P251L KI brains. OmicsIntegrator identified various PPI interaction maps in the optic lobe of tau P251L KI brains, including mitochondrial electron transport chain, larval muscle development, oxidative phosphorylation, tetrahydrofolate metabolic process, nucleus organization, synaptic vesicle exocytosis, lipid modification, cellular response to superoxide and electron transport chain.

Protein interaction networks enriched in the glia of tau P251L KI brains

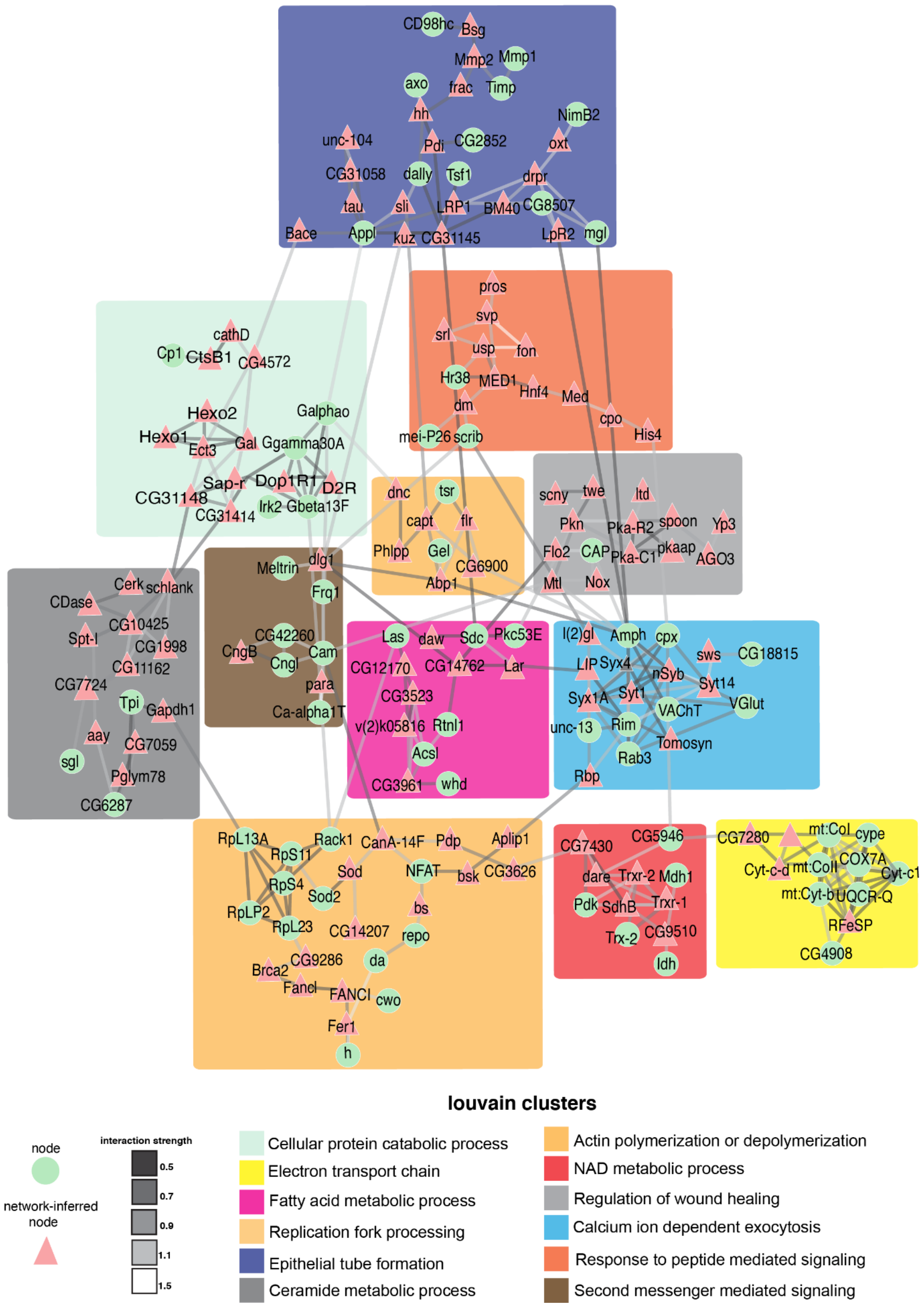

Supplemental Figure S10: Protein interaction networks enriched in glial cells of tau P251L KI brains. OmicsIntegrator identified various PPI interaction maps in glial cells of tau P251L KI brains, including cellular protein catabolic process, electron transport chain, fatty acid metabolic process, replication fork processing, epithelial tube formation, ceramide metabolic process, actin polymerization or depolymerization, NAD metabolic process, regulation of wound healing, calcium ion-dependent exocytosis, response to peptide-mediated signaling, second messenger mediated signaling.

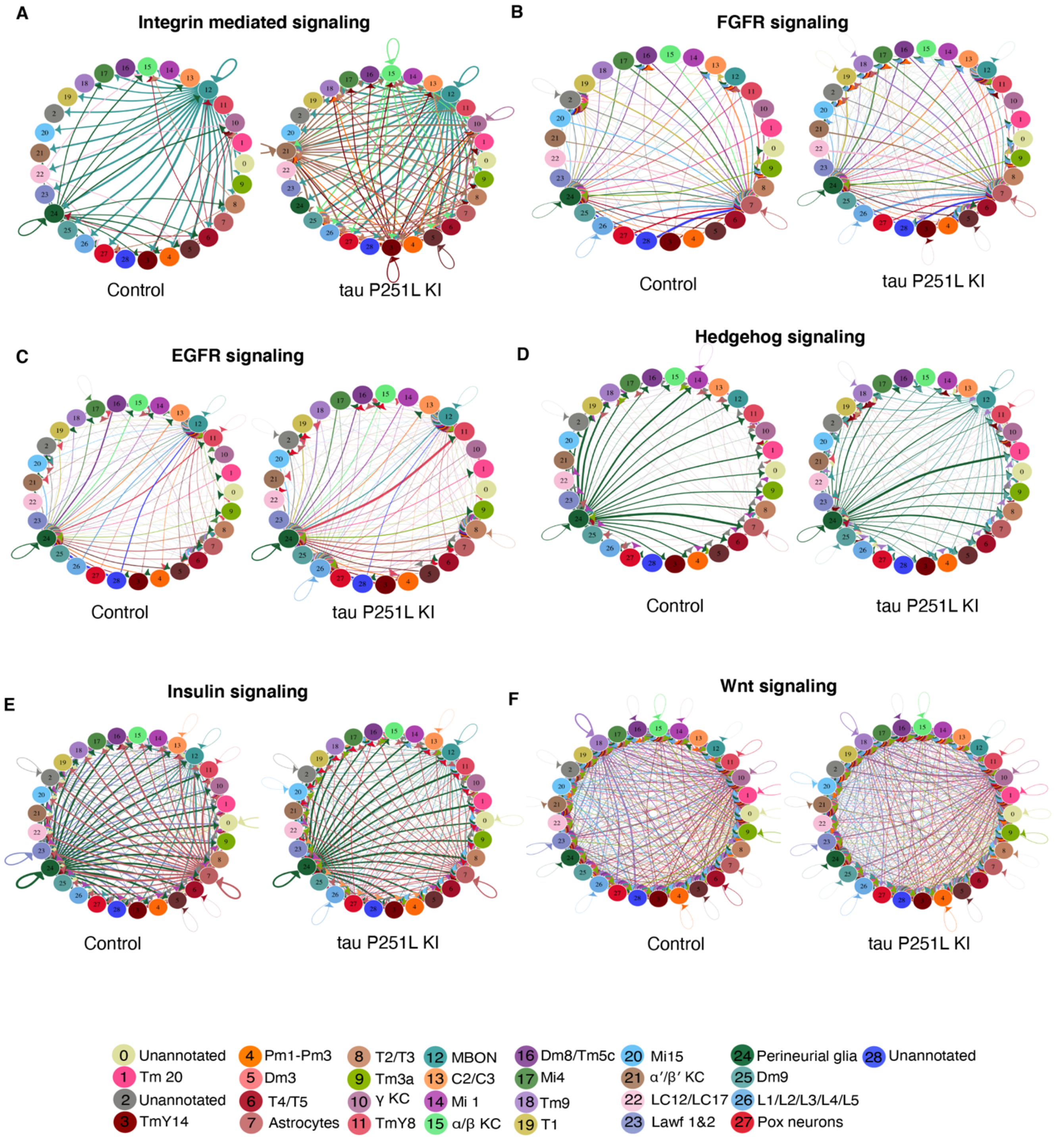
A
Integrin mediated signaling
Control
tau P251L KI
B
FGFR signaling
Control
tau P251L KI
C
EGFR signaling
Control
tau P251L KI
D
Hedgehog signaling
Control
tau P251L KI
E
Insulin signaling
Control
tau P251L KI
F
Wnt signaling
Control
tau P251L KI
0 Unannotated
1 Tm 20
2 Unannotated
3 TmY14
4 Pm1-Pm3
5 Dm3
6 T4/T5
7 Astrocytes
8 T2/T3
9 Tm3a
10 γ KC
11 TmY8
12 MBON
13 C2/C3
14 Mi 1
15 α/β KC
16 Dm8/Tm5c
17 Mi4
18 Tm9
19 T1
20 Mi15
21 α′/β′ KC
22 LC12/LC17
23 Lawf 1&2
24 Perineurial glia
25 Dm9
26 L1/L2/L3/L4/L5
27 Pox neurons
28 Unannotated

Supplemental Figure S11: Compared to control, cell-cell communication analysis of *tau* P251L KI brains reveals a distinctive role of glial and Kenyon cells. Perineurial glial cells actively send ligands of integrin-mediated signaling to other clusters in the control brain, but perineurial don't send these signals in tau P251L KI brains *(A)*. EGFR signaling increases from perineurial glial cells to the other tau P251L KI brain clusters. Further, 4 clusters send ligands of the EGFR signaling in control, but 5 clusters send ligands in tau P251L KI brains *(B)*. Like EGFR signaling, FGFR signaling increases in tau P251L KI brains: 4 clusters send ligands in the control brain, but 8 clusters send ligands in tau P251L KI brains *(C)*. An increase of hedgehog signaling from the perineurial glial cells to other clusters can be observed in tau P251L KI brains. Further, 4 clusters send hedgehog signaling ligands in the control brains, but 5 clusters in tau P251L KI brains *(D).* A decrease of insulin (10 clusters send ligands in the control while 8 clusters send ligands in tau P251L KI brains) and Wnt signaling (decreased communication lines from cluster 18 in tau P251L KI brains) in tau P251L KI brains *(E&F)*.

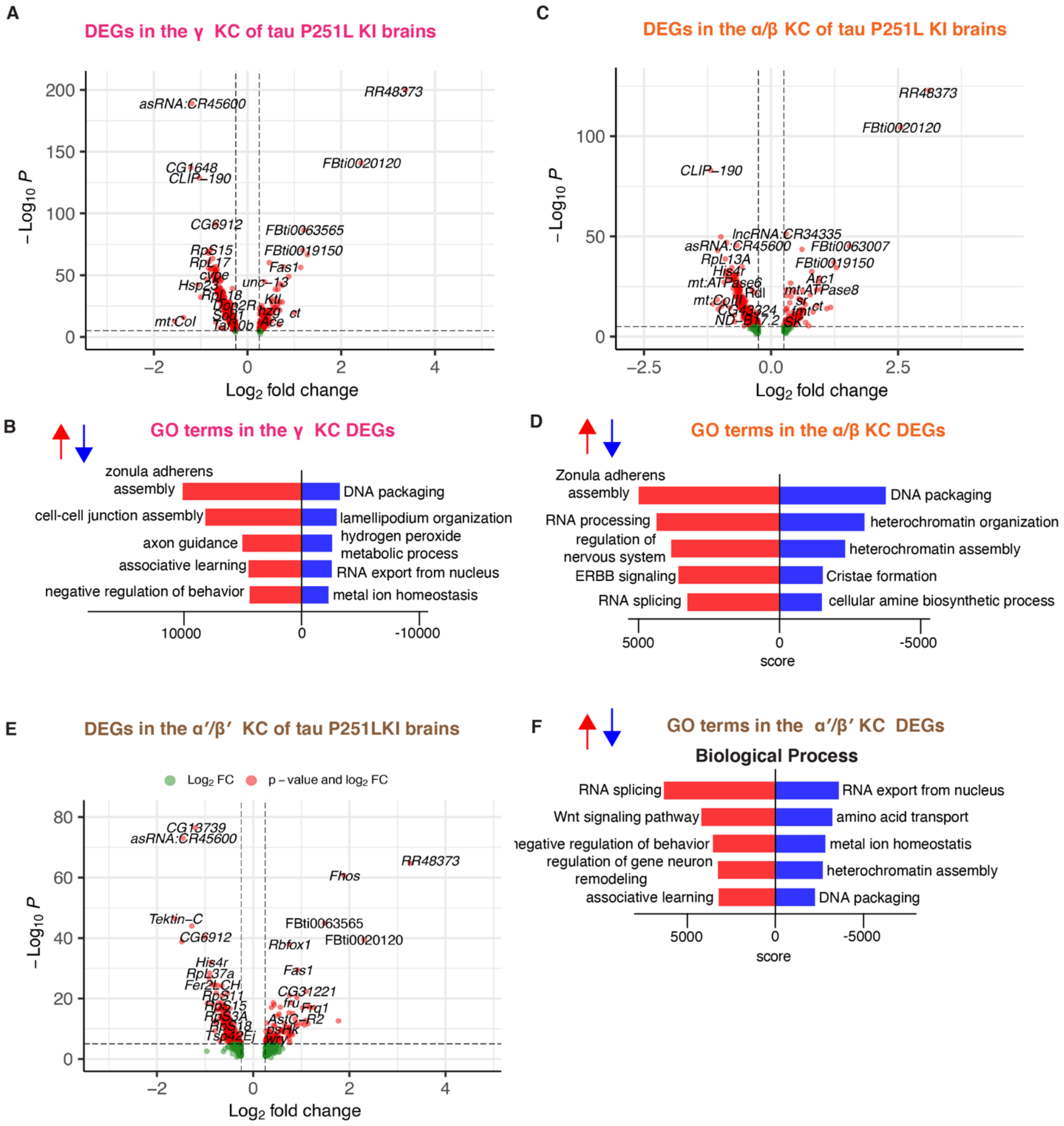
A
DEGs in the γ KC of tau P251L KI brains
−Log10 P
Log2 fold change
RR48373
asRNA:CR45600
FBti0020120
CG1648
CLIP−190
CG6912
FBti0063565
FBti0019150
Fas1
unc−13
mt:CoI
B
GO terms in the γ KC DEGs
zonula adherens assembly
cell-cell junction assembly
axon guidance
associative learning
negative regulation of behavior
DNA packaging
lamellipodium organization
hydrogen peroxide metabolic process
RNA export from nucleus
metal ion homeostasis
10000
0
-10000
C
DEGs in the α/β KC of tau P251L KI brains
−Log10 P
Log2 fold change
RR48373
FBti0020120
CLIP−190
lncRNA:CR34335
asRNA:CR45600
FBti0063007
FBti0019150
D
GO terms in the α/β KC DEGs
Zonula adherens assembly
RNA processing
regulation of nervous system
ERBB signaling
RNA splicing
DNA packaging
heterochromatin organization
heterochromatin assembly
Cristae formation
cellular amine biosynthetic process
5000
0
-5000
score
E
DEGs in the α′/β′ KC of tau P251LKI brains
Log2 FC
p − value and log2 FC
−Log10 P
Log2 fold change
CG13739
asRNA:CR45600
RR48373
Fhos
Tektin−C
CG6912
FBti0063565
Rbfox1
FBti0020120
Fas1
CG31221
F
GO terms in the α′/β′ KC DEGs
Biological Process
RNA splicing
Wnt signaling pathway
negative regulation of behavior
regulation of gene neuron remodeling
associative learning
RNA export from nucleus
amino acid transport
metal ion homeostatis
heterochromatin assembly
DNA packaging
5000
0
-5000
score

Supplemental Figure S12: Differential gene expression and enrichment analysis in the Kenyon cells (KC) of tau P251L KI brains compared to controls. The volcano plot shows differentially up and down-regulated genes in the γ KC cluster *(A)*. Gene Ontology analysis found zonula adherens assembly to be the most common up-regulated and DNA packaging to be the most common down-regulated biological process in the γ KC cluster *(B)* The volcano plot shows differentially up and down-regulated genes in the α/β KC cluster *(C)*. Gene Ontology analysis found zonula adherens assembly to be the most common up-regulated and DNA packaging to be the most common down-regulated biological process in the α/β KC cluster *(D)*. The volcano plot shows differentially up and down-regulated genes in the α'/β' KC cluster *(F)*. Gene Ontology analysis found RNA splicing to be the most common up-regulated and RNA export from the nucleus to be the most common down-regulated biological process in the α'/β' KC cluster *(F)*. All red dots on the volcano plots are significant genes meeting the cutoff of FDR-adjusted p-value < 0.05 and $\log_2$fold-change > 0.25 for up-regulated and < - 0.25 for down-regulated genes, score = log(p)*z.

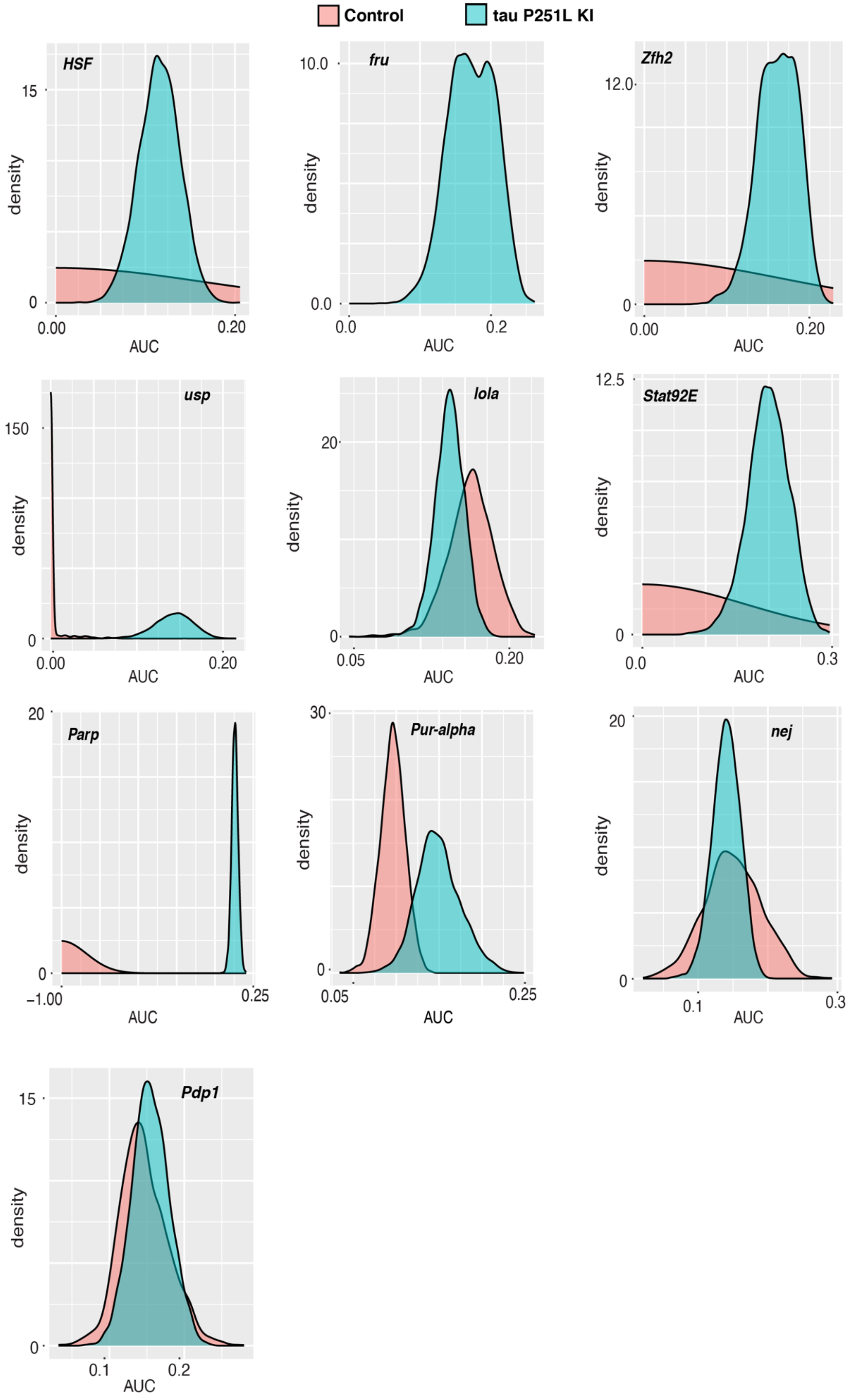
Control
tau P251L KI
HSF
fru
Zfh2
usp
lola
Stat92E
Parp
Pur-alpha
nej
Pdp1
density
AUC

Supplemental Figure S13: Density plots compare the expression of regulons enriched in tau P251L knock-in Kenyon cells compared to controls. The density plots comparing the expression of regulons in the control vs tau P251L knock-in Kenyon cells show that the expression of regulons such as *HSF*, *fru*, *Zfh2*, *usp*, *Stat92E*, and *Parp* is significantly elevated in tau P251L knock-in Kenyon cells compared to controls.

Supplemental References

Becht E, McInnes L, Healy J, Dutertre C-A, Kwok IW, Ng LG, Ginhoux F, Newell EW. 2019. Dimensionality reduction for visualizing single-cell data using UMAP. *Nature biotechnology* **37**: 38-44.

Butler A, Hoffman P, Smibert P, Papalexi E, Satija R. 2018. Integrating single-cell transcriptomic data across different conditions, technologies, and species. *Nature biotechnology* **36**: 411-420.

Chambers JM, Hastie TJ. 2017. Statistical models. In *Statistical models in S*, pp. 13-44. Routledge.

Chen EY, Tan CM, Kou Y, Duan Q, Wang Z, Meirelles GV, Clark NR, Ma'ayan A. 2013. Enrichr: interactive and collaborative HTML5 gene list enrichment analysis tool. *BMC bioinformatics* **14**: 1-14.

Davie K, Janssens J, Koldere D, De Waegeneer M, Pech U, Kreft Ł, Aibar S, Makhzami S, Christiaens V, González-Blas CB. 2018. A single-cell transcriptome atlas of the aging Drosophila brain. *Cell* **174**: 982-998. e920.

Finak G, McDavid A, Yajima M, Deng J, Gersuk V, Shalek AK, Slichter CK, Miller HW, McElrath MJ, Prlic M. 2015. MAST: a flexible statistical framework for assessing transcriptional changes and characterizing heterogeneity in single-cell RNA sequencing data. *Genome biology* **16**: 1-13.

Guo M, Bao EL, Wagner M, Whitsett JA, Xu Y. 2017. SLICE: determining cell differentiation and lineage based on single cell entropy. *Nucleic acids research* **45**: e54-e54.

Hu Y, Tattikota SG, Liu Y, Comjean A, Gao Y, Forman C, Kim G, Rodiger J, Papatheodorou I, Dos Santos G. 2021. DRscDB: a single-cell RNA-seq resource for data mining and data comparison across species. *Computational and Structural Biotechnology Journal* **19**: 2018-2026.

Kuleshov MV, Jones MR, Rouillard AD, Fernandez NF, Duan Q, Wang Z, Koplev S, Jenkins SL, Jagodnik KM, Lachmann A. 2016. Enrichr: a comprehensive gene set enrichment analysis web server 2016 update. *Nucleic acids research* **44**: W90-W97.

Li H, Horns F, Wu B, Xie Q, Li J, Li T, Luginbuhl DJ, Quake SR, Luo L. 2017. Classifying Drosophila olfactory projection neuron subtypes by single-cell RNA sequencing. *Cell* **171**: 1206-1220. e1222.

Li H, Janssens J, De Waegeneer M, Kolluru SS, Davie K, Gardeux V, Saelens W, David FP, Brbić M, Spanier K. 2022. Fly Cell Atlas: A single-nucleus transcriptomic atlas of the adult fruit fly. *Science* **375**: eabk2432.

Liu Y, Li JSS, Rodiger J, Comjean A, Attrill H, Antonazzo G, Brown NH, Hu Y, Perrimon N. 2022. FlyPhoneDB: an integrated web-based resource for cell–cell communication prediction in Drosophila. *Genetics* **220**: iyab235.

Sarkar S, Murphy MA, Dammer EB, Olsen AL, Rangaraju S, Fraenkel E, Feany MB. 2020. Comparative proteomic analysis highlights metabolic dysfunction in alpha-synucleinopathy. *NPJ Parkinsons Dis* **6**: 40.

Street K, Risso D, Fletcher RB, Das D, Ngai J, Yosef N, Purdom E, Dudoit S. 2018. Slingshot: cell lineage and pseudotime inference for single-cell transcriptomics. *BMC genomics* **19**: 1-16.

Team RC. 2016. R: A language and environment for statistical computing. R Foundation for Statistical Computing, Vienna, Austria. *http://www R-project org/*.

Tuncbag N, Gosline SJ, Kedaigle A, Soltis AR, Gitter A, Fraenkel E. 2016. Network-based interpretation of diverse high-throughput datasets through the omics integrator software package. *PLoS computational biology* **12**: e1004879.

Van de Sande B, Flerin C, Davie K, De Waegeneer M, Hulselmans G, Aibar S, Seurinck R, Saelens W, Cannoodt R, Rouchon Q. 2020. A scalable SCENIC workflow for single-cell gene regulatory network analysis. *Nature Protocols* **15**: 2247-2276.
